# Genetic variants including markers from the exome chip and metabolite traits of type 2 diabetes

**DOI:** 10.1038/s41598-017-06158-3

**Published:** 2017-07-20

**Authors:** Susanne Jäger, Simone Wahl, Janine Kröger, Sapna Sharma, Per Hoffmann, Anna Floegel, Tobias Pischon, Cornelia Prehn, Jerzy Adamski, Martina Müller-Nurasyid, Melanie Waldenberger, Konstantin Strauch, Annette Peters, Christian Gieger, Karsten Suhre, Harald Grallert, Heiner Boeing, Matthias B. Schulze, Karina Meidtner

**Affiliations:** 1 Department of Molecular Epidemiology, German Institute of Human Nutrition Potsdam-Rehbruecke, Nuthetal, Germany; 2 German Center for Diabetes Research (DZD), Neuherberg, Germany; 3 Institute of Epidemiology II, Helmholtz Zentrum München, German Research Center for Environmental Health, Neuherberg, Germany; 4 Research Unit of Molecular Epidemiology, Helmholtz Zentrum München, German Research Center for Environmental Health, Neuherberg, Germany; 5 Division of Medical Genetics, Department of Biomedicine, University of Basel, Basel, Switzerland; 6 Department of Genomics, Life and Brain Center, Bonn, Germany; 7 Institute of Human Genetics, University of Bonn, Bonn, Germany; 8 Department of Epidemiology, German Institute of Human Nutrition Potsdam-Rehbruecke, Nuthetal, Germany; 9 Molecular Epidemiology Group, Max Delbrueck Center for Molecular Medicine in the Helmholtz Association (MDC), Berlin-Buch, Germany; 10 Charité – Universitätsmedizin Berlin, Berlin, Germany; 11 DZHK (German Centre for Cardiovascular Research), partner site Berlin, Berlin, Germany; 12 Genome Analysis Center, Institute of Experimental Genetics, Helmholtz Zentrum München, German Research Center for Environmental Health, Neuherberg, Germany; 13 Chair of Experimental Genetics, Center of Life and Food Sciences Weihenstephan, Technische Universität München, Freising-Weihenstephan, Germany; 14 Institute of Genetic Epidemiology, Helmholtz Zentrum München, German Research Center for Environmental Health, Neuherberg, Germany; 15 Department of Medicine I, University Hospital Grosshadern, Ludwig-Maximilians-Universität, Munich, Germany; 16 DZHK (German Centre for Cardiovascular Research), partner site Munich Heart Alliance, Munich, Germany; 17 Institute of Medical Informatics, Biometry and Epidemiology, Chair of Genetic Epidemiology, Ludwig-Maximilians-Universität, Munich, Germany; 18 Department of Physiology and Biophysics, Weill Cornell Medical College in Qatar, Qatar Foundation-Education City, Doha, Qatar

## Abstract

Diabetes-associated metabolites may aid the identification of new risk variants for type 2 diabetes. Using targeted metabolomics within a subsample of the German EPIC-Potsdam study (n = 2500), we tested previously published SNPs for their association with diabetes-associated metabolites and conducted an additional exploratory analysis using data from the exome chip including replication within 2,692 individuals from the German KORA F4 study. We identified a total of 16 loci associated with diabetes-related metabolite traits, including one novel association between rs499974 (*MOGAT2*) and a diacyl-phosphatidylcholine ratio (PC aa C40:5/PC aa C38:5). Gene-based tests on all exome chip variants revealed associations between *GFRAL* and PC aa C42:1/PC aa C42:0, *BIN1* and SM (OH) C22:2/SM C18:0 and *TFRC* and SM (OH) C22:2/SM C16:1). Selecting variants for gene-based tests based on functional annotation identified one additional association between *OR51Q1* and hexoses. Among single genetic variants consistently associated with diabetes-related metabolites, two (rs174550 (*FADS1*), rs3204953 (*REV3L*)) were significantly associated with type 2 diabetes in large-scale meta-analysis for type 2 diabetes. In conclusion, we identified a novel metabolite locus in single variant analyses and four genes within gene-based tests and confirmed two previously known mGWAS loci which might be relevant for the risk of type 2 diabetes.

## Introduction

**Figure 1:**
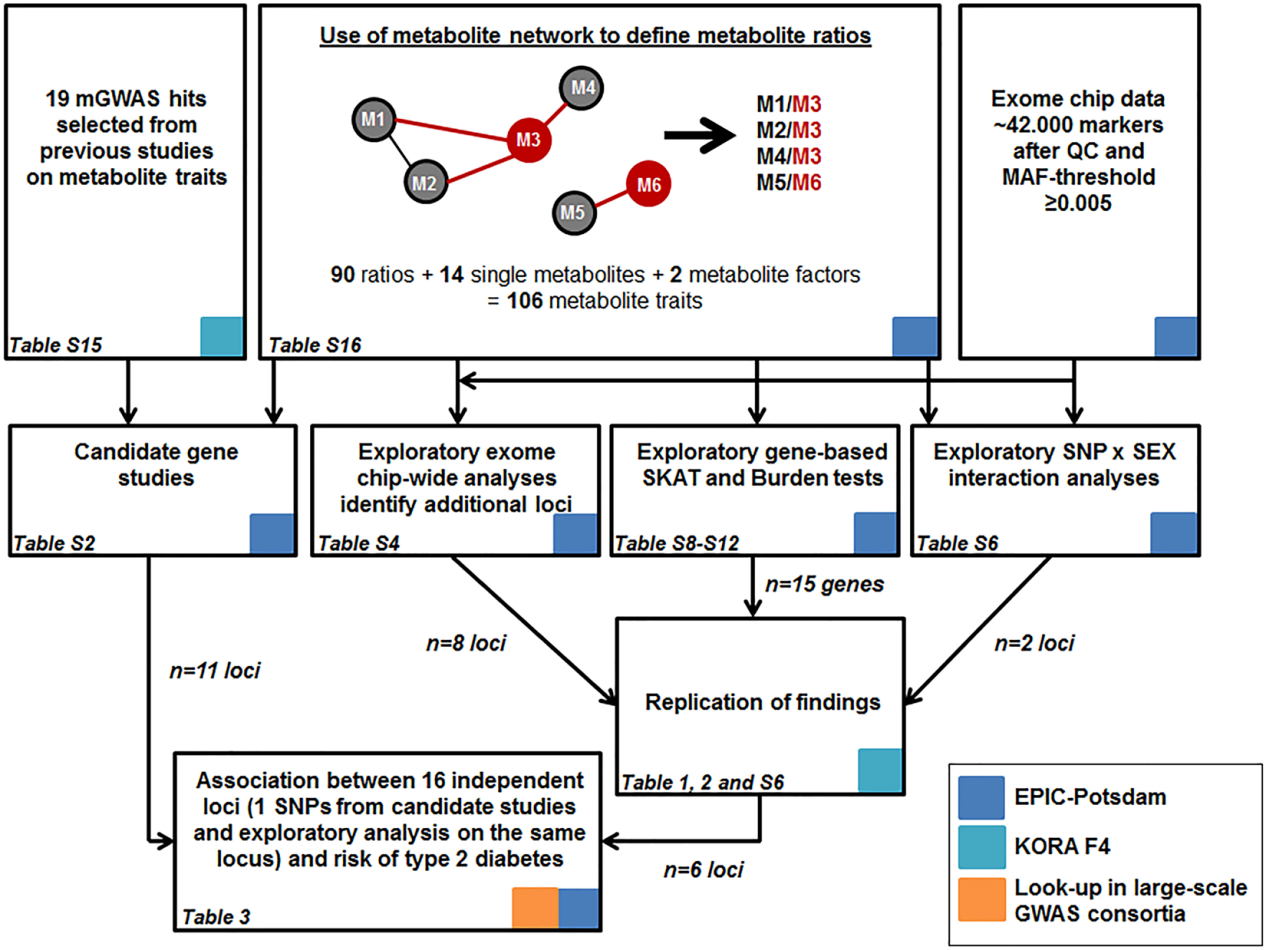
Flowchart of the analytical strategy. The red circles indicate diabetes-associated metabolites and grey circles depict other metabolites. The colored squares indicate data sources used in the different steps of the analysis. GWAS, genome-wide association study; MAF, minor allele frequency; QC, quality control; SNP, single nucleotide polymorphism.

Up to now numerous common risk variants (MAF ≥ 5%) for type 2 diabetes were identified by genome-wide association studies (GWAS)^1, 2^. However, they only explain a small proportion of the population variance^1, 2^. To address this missing heritability, current studies concentrate on rare variants with potentially strong effects. One study identified four low frequency and rare variants which were associated with risk of type 2 diabetes^3^. Furthermore, the CHARGE consortium used exome chip variants from 23 different studies and found a novel association of a low-frequency nonsynonymous variant in the *GLP1R* with type 2 diabetes by an initial exploratory analysis of fasting glucose and insulin and a subsequent analysis of significant results for association with type 2 diabetes^4^. Similarly, metabolites associated with the risk of type 2 diabetes may aid the identification of new type 2 diabetes risk variants^5^. GWAS of metabolomics datasets (mGWAS) identified several so-called genetically influenced metabotypes (GIMs)^6–10^ that show large genetic effect sizes explaining 10–20% of the observed variance in the metabolite traits^11^. In the current study population (EPIC-Potsdam), 14 single metabolites have been identified to be independently associated with risk of type 2 diabetes^12^. Two metabolite factors (principal components) largely explained the variance in metabolite traits and were associated with risk of type 2 diabetes in opposing directions^12^. Within this study, we aim to identify genetic variants which are associated with those previously identified diabetes-related metabolite traits^12^ and test for their association with type 2 diabetes risk. To do so, we firstly, investigated selected known mGWAS loci^7, 8^ for their association with diabetes-related metabolite traits (Fig. 1). Secondly, we conducted an exploratory analysis using HumanExome v1.1 Bead Array data to additionally explore the association of low-frequency (1% < MAF < 5%) and rare genetic variants (MAF < 1%) which were not captured by classical GWAS arrays (Fig. 1). Finally, we analyzed the associations between single genetic variants identified for being associated with diabetes-associated metabolite traits and risk of type 2 diabetes.

## Results

All primary analyses have been carried out within the EPIC-Potsdam study with up to 2932 participants within the diabetes analyses (Fig. 1). Results of exploratory analyses were replicated in the KORA F4 study with up to 2692 participants (Fig. 1). Baseline characteristics of both study populations can be found in Supplementary Table S1.

### Eleven known mGWAS loci were associated with diabetes-related metabolite traits

We analyzed the association of 19 known mGWAS loci with 106 diabetes-associated metabolite traits (Fig. 1). These metabolite traits included single metabolites (n = 14), metabolite ratios defined on the basis of the observed metabolite network (n = 90) and two metabolite factors derived from PCA of diabetes-associated metabolites^12^.

Eleven of the 19 candidate SNPs from previous studies were associated with diabetes-related metabolite traits (P < 4.31 × 10^−5^) in EPIC-Potsdam (Supplementary Table S2). The SNP rs174547 (*FADS1*) was significantly associated with 36 single metabolite traits which primarily originate from the groups of acyl-alkyl-phosphatidylcholines and diacyl-phosphatidylcholines. Further statistically significant associations were found for rs9393903 (*ELOVL2*), rs7156144 (*PLEKHH1*), rs12641551 (*ACSL1*), rs272893 (*SLC22A4*/ *OCTN1*), rs603424 (*SCD*) and phosphatidylcholine ratios. Rs715 (*CPS1*), rs541503 (*PHGDH*) and rs1718306 (*PAH*) were associated with amino acids. Rs11158519 (*SYNE2*), rs364585 (*SPTLC3*) and rs603424 (*SCD*) were related to ratios of sphingomyelins. Previous reports for genotype-by-sex-interactions of rs715 (CPS1) indicating larger effects in women than in men were confirmed in EPIC-Potsdam (Supplementary Table S3)

### Exploratory analysis using exome chip data identified one novel locus for diabetes-associated metabolite traits

We carried out an exploratory analysis of all common (MAF ≥ 5%), low-frequency (1% ≤ MAF < 5%) and rare variants (MAF < 1%) with an allele frequency above 0.5% (n ≈ 42.000) for association with 106 diabetes-related metabolite traits (Fig. 1). The analysis revealed twelve loci that were associated with one or multiple metabolite traits (Supplementary Table S4). As the exome chip includes previously published GWAS hits, some of our results from the exploratory analysis overlap with findings from our candidate gene studies by representing the same locus (*FADS1*, *CPS1*, *SGPP1*/*SYNE2*) or the same SNP (rs12641551(*ACSL1*), rs364585 (*SPTLC3*)). For overlapping results between candidate and exploratory analyses we calculated models including all associated SNPs (from candidate and exploratory analysis) at each locus for the top associated metabolite trait to identify independent signals (Supplementary Table S5). The exome chip variant rs174550 (*FADS1*) showed stronger associations with the PC aa C36:3/PC aa C36:4 ratio than the previously known mGWAS SNP rs174547 on the *FADS1* locus (Supplementary Table S5). An intergenic exome chip variant on chromosome 2 (rs4672596) near the previously identified *CPS1* locus was associated with levels of glycine and the ratio of glycine and serine (Supplementary Table S4). However, the previously identified mGWAS SNP rs715 showed stronger associations in analyses on glycine levels (Supplementary Table S5). One low-frequency variant from the exome chip (rs12881815 located within *SYNE2* with MAF = 4.8%) showed significant associations with two sphingomyelin ratios (SM C16:1/PC aa C28:1 and SM (OH) C22:2/SM (OH) C14:1) (Supplementary Table S4). However, in analyses including all three variants on *SYNE2* (Supplementary Table S5) with the SM C16:1/PC aa C28:1 ratio, the exome chip variant rs7157785 (*SGPP1*) showed stronger effects than rs12881815 (*SYNE2*) and stronger effects than the previously identified mGWAS SNP (rs11158519 (*SYNE2*)).

We aimed to replicate suggestive significant findings from EPIC-Potsdam (P < 1.64 × 10^−7^) in the KORA F4 study. Meta-analysis of results from both studies revealed six significant loci (P < 4.55 × 10^−3^) associated with one or multiple metabolite traits (Table 1). Among them was one novel locus, rs499974 (*MOGAT2*), that was associated with the ratio of PC aa C40:5/PC aa C38:5 (*P* = 6.88 × 10^−15^). The most deleterious variants identified within this study were located in *APOE* (CADD = 30.0) and *REV3L* (CADD = 32.0).

**Table 1:** Exome chip variants associated with metabolite traits at suggestive significance in EPIC-Potsdam and replication in KORA F4.

Metabolite trait	Chr	SNP^b^ (Locus)	*EPIC-Potsdam* ^a^				*KORA F4*				*pooled*		Replicated ^e^	Consequence (GRCH37)	scaled CADD score
			n^c^	allele frequency (coded allele)	Beta (SE)^d^	*p*	n^c^	allele frequency (coded allele)	Beta (SE)^d^	*p*	Beta (SE) ^d^	*p*			
PC aa C42:1/ PC aa C42:0	1	rs41282492 (*CHIA*)	2190	87.9 (A)	0.25 (0.05)	1.01 E-07	2692	87.8 (A)	−0.09 (0.04)	2.78 E-02	0.08 (0.17)	6.41 E-01		Asn45Asp	0.01
Tyrosine/ Methionine	6	rs3204953 (*REV3L*)	2201	85.2 (C)	−0.28 (0.04)	9.80 E-12	2689	84.0 (C)	−0.20 (0.03)	1.49 E-09	−0.24 (0.04)	7.45 E-09	*	Val3064Ile	32
PC aa C36:1/ PC aa C34:1	10	rs10885997 (*PNLIPRP2*)	2201	58.8 (A)	−0.17 (0.03)	5.54 E-08	2692	58.6 (A)	−0.09 (0.02)	1.70 E-05	−0.13 (0.04)	1.46 E-03	*	Synonymous variant	7.74
PC aa C40:5/ PC aa C38:5	11	rs499974 (*MOGAT2*)	2203	81.2 (C)	−0.21 (0.04)	2.25 E-08	2692	81.3 (C)	−0.18 (0.03)	6.97 E-08	−0.19 (0.03)	6.88 E-15	*	downstream gene variant	8.66
PC ae C44:6/ PC aa C42:1	11	rs10790162 (*BUD13*)	2203	6.70 (A)	−0.32 (0.06)	1.57 E-07	2691	7.56 (A)	−0.06 (0.05)	2.51 E-01	−0.19 (0.13)	1.53 E-01		intron variant	6.55
SM C16:1/ PC aa C28:1	14	rs7157785 (*SGPP1*)	2203	83.6 (G)	0.49 (0.04)	1.45 E-35	2691	82.6 (G)	0.40 (0.03)	2.27 E-40	0.45 (0.04)	2.39 E-24	*	regulatory region variant	1.91
SM (OH) C22:2/ SM C24:0	14	rs7157785 (*SGPP1*)	2203	83.6 (G)	−0.22 (0.04)	5.46 E-10	2692	82.6 (G)	−0.24 (0.03)	6.40 E-15	−0.23 (0.02)	9.22 E-24	*	regulatory region variant	1.91
SM (OH) C22:2/ SM (OH) C14:1	14	rs7157785 (*SGPP1*)	2202	83.6 (G)	0.42 (0.04)	2.79 E-27	2692	82.6 (G)	0.37 (0.03)	4.21 E-28	0.40 (0.03)	7.09 E-55	*	regulatory region variant	1.91
SM (OH) C22:2/ SM (OH) C22:1	14	rs7157785 (*SGPP1*)	2202	83.6 (G)	−0.30 (0.04)	6.00 E-15	2691	82.6 (G)	−0.27 (0.03)	9.89 E-17	−0.28 (0.02)	7.52 E-31	*	regulatory region variant	1.91
	19	rs7412 (*APOE*)	2202	91.4 (C)	−0.27 (0.05)	1.30 E-07	2688	91.5 (C)	−0.14 (0.04)	1.76 E-03	−0.20 (0.06)	1.48 E-03	*	Arg202Cys	30
PC aa C36:3/ PC aa C34:3	16	rs1136001 (*NTAN1*)	2201	67.0 (G)	0.18 (0.03)	5.58 E-09	2690	69.0 (G)	0.14 (0.03)	3.29 E-07	0.16 (0.02)	1.18 E-14	*	His283Asn	0.81

Chr, chromosome; SE, standard error; CADD, Combined Annotation Dependent Depletion. ^a^only sub-cohort; ^b^gene variants are reported on the forward strand of NCBI build 37; ^c^metabolite outliers (±4SD) were excluded; ^d^metabolites (µmol/L) were ln-transformed and standardized, effect estimates are adjusted for age and sex; ^e^significance threshold: 0.05/11 tests = 4.55 × 10^−3^.

As previous studies observed that SNP-metabolite associations might be different between women and men, we analyzed SNP-by sex interactions (Fig. 1). Although two variants indicated significant interaction with sex in EPIC-Potsdam, these findings could not be confirmed in KORA F4 (Supplementary Table S6).

**Figure 2:**
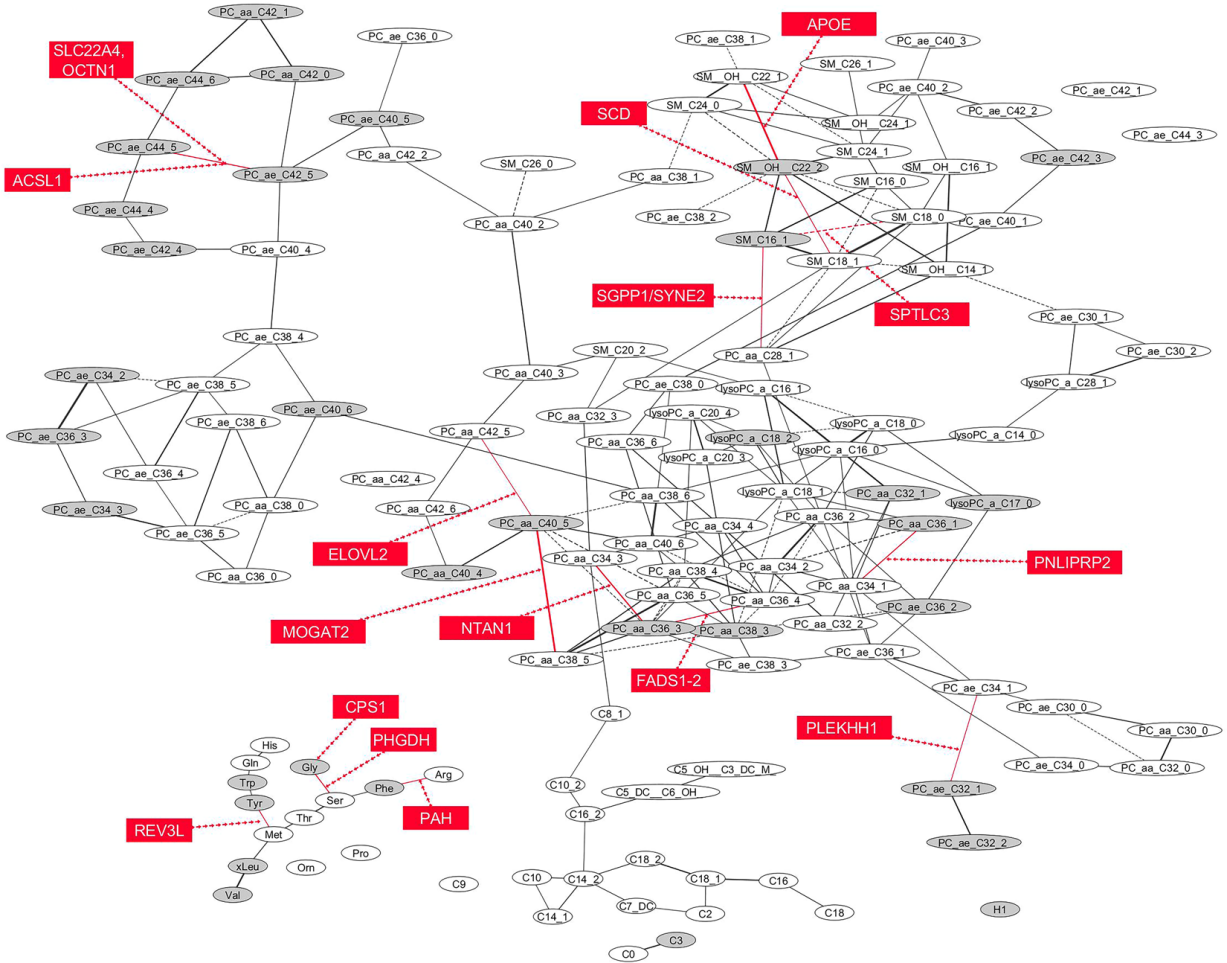
Network structure of metabolites within EPIC-Potsdam and related genetic variants. 34 diabetes associated metabolites are depicted in grey elipses; sixteen identified genetic variants associated with diabetes-related metabolite traits are depicted in red rectangles (only the top associated metabolite trait is depicted for each locus); solid line indicates direct association between metabolites; dashed line indicates inverse association between metabolites; GGM network was adapted from the publication by A. Floegel *et al*.^53^ and drawn by using Cytoscape Software v3.2.1^66^.

The relation of the identified SNPs with the metabolic network structures is depicted in Fig. 2.

### Biologic pathway annotations

Not all variants could be linked to specific pathways within the KEGG database (Supplementary Table S7). However, with regard to the annotated gene functions plausible metabolic pathways were found for most variants. For example, *FADS1* and *FADS2* were linked to “Biosynthesis of unsaturated fatty acids” and *FADS2* was associated with “alpha-Linolenic acid metabolism” and “PPAR signalling pathway”. *MOGAT2* was connected to *“*Glycerolipid metabolism” and “Fat digestion and absorption”.

### Gene-based analyses identified four genes associated with metabolite traits

Single SNP analyses of low-frequency and rare variants are usually considered underpowered in studies of moderate sample size like ours^13^. Therefore, we conducted two gene-based analyses to enhance the power of our study (Fig. 1). We identified ten genes associated with metabolite traits in SKAT or burden tests (Supplementary Table S8). However, only three genes (*GFRAL*, *BIN1*, *TFRC*) showed nominal significant associations (P < 0.05) with metabolite traits within KORA F4 (Table 2). The association for *GFRAL* included three low-frequency variants (rs147652095, rs115053739, rs146300118; all with MAF = 1.8%) while *BIN1* and *TFRC* included only common variants (Supplementary Table S9). In a second step, we considered functional annotation and restricted the gene-based analysis to those exome chip variants which are predicted to be damaging (see Methods section). This analysis revealed six different genes in EPIC-Potsdam (Supplementary Table S10) of which, only one (*OR51Q1*) including two low-frequency variants (rs151161477 with MAF = 1.21% and rs58283839 with MAF = 1.57%) (Supplementary Table S11) was replicated in within KORA F4 (Table 2). By excluding the top variants from the gene-based analyses (see Methods section) we found that multiple low-frequency variants within *GFRAL* are involved within the association, while in *OR51Q1*, the association is driven by the top low-frequency variant (rs58283839) (Supplementary Table S12). However, none of the single SNPs within the genes was significantly associated with metabolite traits individually (Supplementary Tables S9 and S11).

**Table 2:** Gene-based association with metabolite traits using SKAT and burden test in EPIC-Potsdam and replicated in KORA F4.

Metabolite trait	Gene	EPIC-Potsdam^a^				KORA F4^a^				
		Nr. of common variants^b^	Nr. of rare variants^b^	P_SKAT-C_	P_burden-C_	Nr. of common variants^b^	Nr. of rare variants^b^	P_SKAT-C_	P_burden-C_	Replicated in KORA F4 (p < 0.05)
PC aa C42:1/PC aa C42:0	*GFRAL*	6	0	1.54E-05^c^	2.15E-06^c^	6	0	1.02E-02	4.32E-03	*
SM (OH) C22:2/SM C18:0	*BIN1*	2	0	7.98E-05^c^	1.50E-06^c^	2	0	1.96E-02	3.70E-01	*
SM (OH) C22:2/SM C16:1	*TFRC*	3	0	1.66E-06^c^	7.53E-04^c^	3	0	2.30E-02	9.08E-02	*
H1	*OR51Q1* ^d^	2	1	8.85E-06^e^	9.62E-06^e^	2	1	1.47E-02	1.38E-02	*

^a^EPIC-Potsdam: n = 2200–2202, KORA F4: n = 2688–2692; analyses were adjusted for age and sex. ^b^Rare variants with MAF ≤ $\,\frac{1}{\sqrt{2n}}$ (≤0.015 _EPIC-Potsdam_ or ≤0.014 _KORA F4_). ^c^Significance threshold was defined as P < 0.05/[number of genes with >1 variants (ranging from 7243 to 7332)] = 6.8 × 10^−6^ to 6.9 × 10^−6^. ^d^Identified by restricting the analysis on potentially damaging variants. ^e^Significance threshold was defined as P < 0.05/[number of genes with >1 variants (ranging from 1449 to 1492)] = 3.4 × 10^−5^ to 3.5 × 10^−6^.

### Identified genetic variants were weakly associated with risk of type 2 diabetes and other traits

Among the genes identified to be associated with diabetes-related metabolite traits by gene-based analyses, none was associated with type 2 diabetes in gene-based analyses (Supplementary Table S13).

Based on the single SNP analyses, 16 independent loci could be identified from the candidate SNP analyses and exploratory analyses (Fig. 1).

None of the 16 SNPs associated with diabetes-related metabolic traits (predicted functions are summarized in Supplementary Table S14) was additionally associated with type 2 diabetes within the EPIC-Potsdam study (Table 3). Nevertheless, nine of them (rs541503 (*PHGDH*), rs715 (*CPS1*), rs272893 (*SLC22A4*/*OCTH1*), rs9393903 (*ELOVL2*), rs3204953 (*REV3L*), rs603424 (*SCD*), rs174550 (*FADS1*), rs499974 (*MOGAT2*), rs7157785 (*SGPP1*)) showed nominally significant association with diabetes in published data from bigger GWAS consortia for type 2 diabetes (up to 110.452 individuals) or exome array analyses (up to 75.670 individuals) of which two (rs174550 (*FADS1*) and rs3204953 (*REV3L*)) were significant after correction for multiple testing by FDR (Table 3). For only four out of the nine variants (rs541503 (*PHGDH*), rs715 (*CPS1*), rs603424 (*SCD*), rs499974 (*MOGAT2*)) the expected effect was directional consistent with the one which was actually observed within the DIAGRAM consortium or the GoT2D consortium^2, 14^, resulting in a directional consistency of 44.4% (*P* = 1) in the binomial test. In addition to the association with type 2 diabetes, one variant (rs174550 (*FADS1*)) was significantly associated with fasting glucose based on a large-scale meta-analysis of the MAGIC (n = 58.074)^15^ and two (rs715 (*CPS1*), rs1136001 (*NTAN1*)) were significantly associated with BMI within large-scale GWAS meta-analysis of the GIANT consortium (n = 253.288)^16^ (Table 3).

**Table 3:** HRs (95% CI) for type 2 diabetes of genetic variants in EPIC-Potsdam and look-up in other consortia for type 2 diabetes, fasting glucose and BMI.

Chr	SNP	Locus	MAF in % (minor allele)	n (n_cases_)	Model 1 HR (95% CI)	Model 2 HR (95% CI)	Observed association for type 2 diabetes OR (p-value)^a^	Expected direction of the diabetes association ^h^	Match between expected and observed direction	Association fasting glucose: beta (p-value)^a^	Association BMI: beta (p-value)^a^
1	rs541503	*PHGDH*	37.5 (C)	2893 (753)	0.92 0.81–1.04)	0.91 (0.78–1.06)	0.89 (0.039)^b^	**↓**	yes	−0.003 (0.481)	−0.004 (0.330)
2	rs715	*CPS1*	30.1 (C)	2886 (749)	0.98 (0.86–1.12)	0.93 (0.79–1.08)	0.94 (0.035)^c^	↓	yes	0.007 (0.276)	0.022 (7.13E-06*)
4	rs12641551	*ACSL1*	31.9 (G)	2891 (758)	1.02 (0.90–1.17)	1.02 (0.88–1.18)	0.90 (0.104)^b^	↓	yes	—	—
5	rs272893	*SLC22A4, OCTN1*	38.4 (A)	2891 (758)	1.02 (0.90–1.15)	1.11 (0.97–1.27)	0.93 (0.041)^d^	↓	yes	−0.008 (0.040)	0.004 (0.269)
6	rs9393903	*ELOVL2*	24.5 (A)	2932 (763)	0.99 (0.86–1.15)	0.98 (0.83–1.16)	0.97 (0.040)^e^	↑	no	0.001 (0.816)	0.004 (0.350)
6	rs3204953	*REV3L*	14.7 (A)	2891 (758)	0.94 (0.78–1.12)	0.94 (0.76–1.18)	0.88 (0.0008*)^c^	↑	no	−0.007 (0.199)	−0.002 (0.640)
10	rs603424	*SCD*	18.7 (A)	2892 (759)	1.06 (0.91–1.24)	0.99 (0.82–1.19)	1.07 (0.047)^c^	↑	yes	0.010 (0.083)	−0.002 (0.733)
10	rs10885997	*PNLIPRP2*	41.2 (G)	2891 (758)	0.96 (0.85–1.09)	1.01 (0.88–1.16)	1.04 (0.259)^d^	↑	yes	0.001 (0.787)	0.0004 (0.9203)
11	rs174550	*FADS1*	33.5 (G)	2891 (758)	0.93 (0.82–1.06)	0.98 (0.85–1.13)	0.95 (0.003*)^f^	↑	no	−0.021 (1.48E-8*)	0.003 (0.426)
11	rs499974	*MOGAT2*	18.9 (A)	2891 (758)	1.14 (0.97–1.34)	1.07 (0.89–1.28)	1.03 (0.034)^e^	↑	yes	0.002 (0.716)	−0.006 (0.140)
12	rs1718306	*PAH*	39.9 (T)	2902 (754)	1.01 (0.89–1.15)	0.91 (0.78–1.05)	1.04 (0.13)^c^	↑	yes	0.004 (0.273)	0.004 (0.357)
14	rs7156144	*PLEKHH1*	42.5 (A)	2860 (744)	1.10 (0.96–1.25)	1.06 (0.92–1.23)	1.04 (0.097)^c^	↑	yes	−0.003 (0.506)	0.001 (0.842)
14	rs7157785	*SGPP1*	16.4 (A)	2891 (758)	1.05 (0.90–1.23)	1.08 (0.90–1.30)	1.04 (0.029)^e^	↓	no	0.011(0.047)	0.002 (0.767)
16	rs1136001	*NTAN1*	33.1 (A)	2891 (758)	0.89 (0.78–1.01)	0.89 (0.77–1.04)	0.98 (0.083)^e^	↓	yes	−0.001 (0.903)	−0.013 (0.002*)
19	rs7412	*APOE*	8.60 (A)	2891 (758)	1.08 (0.87–1.33)	0.93 (0.72–1.21)	1.09 (0.371)^d^	↓	no	—	0.018 (0.075)
20	rs364585	*SPTLC3*	38.1 (A)	2891 (758)	0.91 (0.81–1.03)	1.04 (0.90–1.20)	0.95 (0.169)^g^	↑	no	−0.004 (0.239)	−0.003 (0.401)

SNP, single nucleotide polymorphism; Chr, chromosome; MAF, minor allele frequency; HR, hazard ratio; CI, confidence interval; OR, odds ratio. Only genetic variants which could be replicated within KORA F4 were included. Model 1 is stratified for age at baseline and adjusted for sex; Model 2 is further adjusted for waist circumference. ^a^Results for type 2 diabetes were looked up at http://www.type2diabetesgenetics.org on the 21.07.2016^14^; beta estimates for fasting glucose are from MAGIC GWAS data^15^; beta estimates for BMI are from GIANT GWAS data^16^, strongest type 2 diabetes association reported within: ^b^GoT2D WGS, ^c^GoT2D WGS + replication, ^d^GWAS SIGMA, ^e^DIAGRAM, ^f^82k exome chip, ^g^SIGMA exome chip analysis. ^h^Expected direction was defined based on the sign of the product between the SNP-metabolite trait association and the metabolite trait-type 2 diabetes association (using cox-regression models adjusted for age and sex); ↑ Indicates a positive association, ↓ Indicates an inverse association. *Significant after correction for false discovery rate^62^.

## Discussion

Within this study, we investigated the contribution of genetic variants on diabetes-related metabolic traits to get a deeper insight into the underlying biological processes and to identify novel risk variants for this polygenic disease. We tested findings from previous GWAS and found associations with diabetes-associated metabolites for variants located in *FADS1*, *ELOVL2*, *PLEKHH1*, *SPTLC3*, *ACSL1*, *SCD*, *SLC22A4*, *OCTN1*, *PHGDH* ^7^; *CPS1* and *PAH* ^8^. Furthermore, our exploratory analysis identified additional genetic variants from the exome chip, which were located in *REV3L*, *PNLIPRP2*, *NTAN1*, *APOE*, *MOGAT2*, and *SGPP1* and of which one locus (*MOGAT2*) was novel for metabolite traits.

Using diabetes-associated metabolites as intermediate phenotypes provides the possibility to investigate biomarkers which are more proximal to genes and biological pathways than complex disease endpoints, ensuring higher statistical power to detect genetic associations^6^.

To deal with multiple testing issues, we selected a reduced panel of metabolite traits using their already published network structure^17^. This approach further reduces the number of outcomes to be tested compared to studies which used all possible ratios - independent of their correlation structure. Although varying heritability estimates have been found for the analyzed diabetes-associated metabolites traits ranging from low (h^2^ = 3%) for phenylalanine^18^ to moderately to high (33% ≤ h^2^ ≤ 59%)^18^ or even higher e.g. glycine (h^2^ = 70%)^19^, individual metabolic profiles are long-term conserved^20^, supporting their suitability for studying pathways underlying disease development.

Despite the smaller sample size in our study compared to one meta-analysis which analyzed Biocrates metabolites in 7,478 individuals^9^, we identified one novel locus rs499974 (*MOGAT2*) which was related to a ratio between two diacyl-phosphatidylcholines (PC aa C40:5 and PC aa C38:5). This variant was previously mainly shown to be associated with lipid traits (HDL-c, total cholesterol, triglycerides)^21^. Probably due to the limited power of our analysis, none of the single SNP findings could be related to incident type 2 diabetes in EPIC-Potsdam. Nevertheless, two SNPs (*FADS1*, *REV3L*) which were associated with metabolite traits were significant in bigger consortia for type 2 diabetes, such as DIAGRAM^2^ or GoT2D^14^. Furthermore, according to data from the MAGIC consortium^15^, *FADS1* was related to fasting glucose. In addition, we show that the rs174550 (*FADS1*) is stronger related to metabolite traits than the previously known mGWAS hit (rs174547; LD with rs174550: r² = 1; D’ = 1) and remained significantly associated with a ratio of phosphatidylcholines in analyses containing both SNPs. On a functional level, we cannot consider one or another as being more relevant for diabetes risk as the CADD scores are low for both variants (rs174550: CADD = 3.57; rs174547: CADD = 6.23). A recent study identified a functional multiallelic variant (rs174557; LD with rs174550: r² = 0.778; D′ = 1) located in an AluYe5 element in intron 1 of *FADS1* which affects two transcription factor binding sites with opposing effects on *FADS1* expression^22^. This newly described variant may represent the causal variant at the *FADS1* locus. The *FADS1-2* locus is linked to fatty acid metabolism and biosynthesis of unsaturated fatty acids. Fatty acids itself as well as the fatty acid desaturases which catalyse the desaturation of fatty acids are known to be associated with type 2 diabetes risk^23^. Variants within *FADS1* and *FADS2* encoding the fatty acid Δ5 desaturase and Δ6 desaturase, respectively, are located in a gene family (*FADS1-2-3*) and belong to the most frequently identified mGWAS hits in previous studies showing associations with levels of phosphatidylcholines and sphingolipids^6, 7, 24^. In EPIC-Potsdam, the minor allele of the variant rs174546 within *FADS1* (LD with present *FADS1* variant rs174550: r² = 1; D = 1) was associated with lower Δ5 desaturase and Δ6 desaturase activity^23^. While a genetically-determined higher Δ5 desaturase activity tended to be associated with lower type 2 diabetes risk after adjustment for the other estimated desaturase activity, a genetically-determined high Δ6 desaturase activity was significantly associated with higher risk^23^. As this gene cluster is characterized by high LD, it is difficult to assign effects of single genetic variants located at this locus to one or another desaturase activity. Therefore, observed associations for *FADS1* in the present study might be attenuated by confounding by LD of *FADS2* and vice versa.

The second finding was a missense variant *REV3L* (rs3204953, encoding p. Val3064Ile) belonging to the 0.1% most deleterious variants within the human genome (CADD = 32.0)^25^ which was associated with a ratio between tyrosine and methionine and showed significant association with type 2 diabetes in databases after correction for multiple testing. While this variant is common among Europeans (MAF = 17%), it is rather rare in other populations: Africans (MAF = 0.005), East Asians (MAF = 0.001)^26^.

According to the PhenoScanner database^27^, rs3204953 was associated with alanine to tyrosine ratio (p = 2.98 × 10^−11^)^28^, schizophrenia (0.07 for the effect allele (T); p = 2.14 × 10^−5^)^29^ and age at menopause (beta = 0.15 for the effect allele (T); p = 1.10 × 10^−7^)^30^ in previous GWAS. The latter trait is itself related to type 2 diabetes^31^. On the one hand, factors such as sex-hormones are discussed in this context^32^, on the other hand, early menopause might represent a marker for premature aging^31^ as it has been related to DNA damage repair^30^. In this context, *REV3L* encoding the REV3L (REV3 Like, DNA Directed Polymerase Zeta Catalytic Subunit), a specialized DNA polymerase^33^, was linked to the Fanconi anemia pathway in the KEGG database representing an essential pathway for the DNA repair of interstrand cross-links^34^. More specifically, this enzyme is involved in the translesion DNA synthesis, one essential step within the Fanconi anemia pathway, which represents one of the cellular mechanisms for DNA damage tolerance or post-replication repair^34^. For example, Singh *et al*. could show that the human REV3L maintains the integrity of the mitochondrial genome by affecting the mtDNA metabolism and that inactivation of Rev3 leads to mitochondrial dysfunction^35^. There is quite some evidence that DNA damage induced by oxidative stress is relevant in type 2 diabetes and might additionally represent a mechanistic link with cancer^36^; however, whether genetic markers in *REV3L* might play a role in this context needs to be studied.

We applied gene-based tests which have been shown to be better powered than single variant analyses^13^ and a decreased number of tests is carried out by grouping variants based on genes. In these analyses, we identified associations between metabolite outcomes and four genes (*GFRAL*, *BIN1*, *TFRC* and *OR51Q1*). However, none of the identified genes was linked to type 2 diabetes; neither in EPIC-Potsdam nor in previous studies. Although we found associations with the genes and metabolite traits, it is striking that none of the single variants located in the genes showed any association with the metabolites of interest which further impedes interpretation. Another issue is that compared to our single SNP findings, replication of the gene-based results in KORA F4 was less successful and some of the replicated genes included only common variants. Although, we checked the allele frequencies between both cohorts; still, some minor differences might have affected the gene-based results.

Even though our study identified some interesting candidates which were linked to relevant pathways of type 2 diabetes, all identified SNPs in the single SNP analysis were common variants and no low-frequency or rare variants were identified, which is plausible due to the limited power of our analysis.

In general, SNPs can be used to assess the causality of observed associations between biomarkers and complex traits such as type 2 diabetes by Mendelian Randomisation studies. However, Mendelian randomisation studies rely on a number of assumptions of which one is that “the genotype is associated with the outcome through the studied exposure only”^37^ or simply said that there are no pleiotropic effects of the SNP. However, many of the variants in our study, e.g. *FADS1*, *SPTLC3* and *APOE* were associated with different metabolite traits in addition to other biomarkers of lipid metabolism. The observational design of our study does not allow to discriminate if those different effects are all in a causal chain or related via independent physiological mechanisms. Therefore, we did not investigate causal SNP effects of the analysed genetic variants and type 2 diabetes by Mendelian Randomization. However, if we compare our observations to the assumptions of Mendelian randomization, we have to acknowledge that our observations would be more in line with a non-causal relationship between specific metabolites such as certain ratios of diacyl-phosphatidylcholines (PC aa C36:3/PC aa C36:4 or PC aa C38:3/PC aa C38:4) and tyrosine to methionine ratio and type 2 diabetes, since expected and observed effect direction were inconsistent for SNPs in *FADS1* and *REV3L*. Similarly, a recent study^5^ found inconsistent effect directions for two of four nominal significant variants in DIAGRAM data^1^ using intermediate metabolite traits to determine an expected direction of the SNP-diabetes association. However, with regard to *FADS1*, we have only tested the most significant metabolite ratios for their association with type 2 diabetes. Hence, other significant ratios might show diabetes associations with directional consistency.

In summary, we identified a novel metabolite locus (*MOGAT2*) in single variant analyses and four genes (*GFRAL*, *BIN1*, *TFRC*, *OR51Q1*) within gene-based tests and could show that two previously known mGWAS loci (*FADS1* and *REV3L*) might be relevant for the risk of type 2 diabetes. Our findings do not clearly support the idea that specific diabetes-associated metabolite ratios (PC aa C36:3/PC aa C36:4, PC aa C38:3/PC aa C38:4 and tyrosine/methionine) may be causal traits for type 2 diabetes, but rather show that shared genetic influences on diabetes-related metabolite traits and type 2 diabetes itself exist.

## Materials and Methods

### Study population

#### EPIC-Potsdam study

The European Prospective Investigation into Cancer and Nutrition (EPIC)-Potsdam study consists of 27,548 participants recruited between 1994 and 1998 from the general population in Potsdam and surroundings^38^. The baseline examination involved a personal interview including questions on prevalent diseases, a self-administered questionnaire on socio-economic and lifestyle characteristics, interviewer-conducted anthropometric measurements and a blood sample collection^39^. We used a prospective case-cohort nested within the EPIC-Potsdam study, described in detail previously^40^. Briefly, a sub-cohort of 2,500 individuals was randomly selected from 26,444 participants who provided blood samples at baseline. Additionally, 849 incident type 2 diabetes cases were identified in the full cohort of whom 820 cases provided blood samples (Supplementary Fig. S1).

Participants with missing or implausible data on metabolite measurements (described in detail within the metabolite measurements section), prevalent diabetes, or participants with uncompleted follow-up were excluded, leaving 2283 individuals for analyses in the sub-cohort. Similar exclusion criteria were applied for type 2 diabetes cases, leaving 784 incident cases for analysis. Because the sub-cohort is representative of the full cohort at baseline, the sub-cohort included 73 individuals who developed incident type 2 diabetes during follow-up. Exclusions due to missing genotype data or outlying metabolite values were done separately in each analysis.

#### Cooperative Health Research in the Region of Augsburg study (KORA F4)

KORA (Cooperative Health Research in the Augsburg Region) is a research platform of independent population-based health surveys and subsequent follow-up examinations of community-dwelling adults living in the region of Augsburg in Southern Germany. Study design, sampling method and data collection have been described in detail elsewhere^41^. The KORA S4 survey (1999 to 2001) comprised 4,261 participants, 25 to 74 years old^42^. Of these, 3,080 subjects participated in the follow-up examination KORA F4 (2006 to 2008)^43^. The present study is based on a subsample of 2,818 participants of KORA F4 with Biocrates metabolomics data available.

#### Ethics statement

All participants provided written informed consent. The EPIC-Potsdam study was approved by the ethics committee of the State of Brandenburg, Germany and the KORA study was approved by the ethics committee of the Bavarian medical association, Germany. All procedures were in accordance with the ethical standards of the institutional and/or national research committee and with the 1964 Helsinki declaration and its later amendments or comparable ethical standards.

### DNA-extraction, genotyping and quality control

#### EPIC-Potsdam

The DNA has been extracted from buffy coats using the chemagic DNA Buffy Coat Kit special on a Chemagic Magnetic Separation Module I (PerkinElmer Chemagen technologies, Baesweiler, Germany) according to the manufacturer’s manual.

The selection of candidate SNPs (Supplementary Table S15) was based on previous studies available from the literature which were conducted within the KORA F4 study^7, 8^. Illig *et al*.^7^ reported 15 GWAS hits for metabolite traits which showed associations with the 14 diabetes-associated metabolite traits from EPIC-Potsdam. However, one finding from Illig *et al*.^7^ (rs7094971; *SLC16A9*) which was exclusively associated with phenylalanine levels and showed only a small explained variance in the linear model of 0.10 was not selected for our analysis. As Shin *et al*.^8^ found a stronger candidate for glycine levels, the hit reported by Illig *et al*.^7^ (rs2216405; *CPS1*) was replaced by the SNP rs715 (*CPS1*). Additional five candidates for glycine (n = 4) and phenylalanine (n = 1) levels were selected from Shin *et al*.^8^ based on a predefined significance threshold of <10^−6^. If more than one SNP was associated on a specific locus, the one showing the lowest p-value while considering LD structures between the variants was selected. By doing so we ended up with 19 candidate SNPs for the present analysis. If neither the respective variant nor a proxy SNP was present on the exome chip, the variants were genotyped within EPIC-Potsdam by KBioscience¸ Teddington, UK (http://www.kbioscience.co.uk) using KASP SNP genotyping system.

The Illumina HumanExome v1.1 Bead Array^44^ was used for genotyping of type 2 diabetes cases and the sub-cohort. Genotyping was performed in the Life and Brain Center in Bonn, Germany. Genotype calling and quality control (QC) were carried out using Illuminas GenomeStudio v2011.1 software suite. To improve genotype calling, all single nucleotide variants were re-clustered based on a cluster file from genotyping of 23000 women in the Women’s Genome Health Study^45^ genotyped with the Illumina HumanExome v1.1 Bead Array. This cluster file was generated according to the CHARGE Best Practices and Joint Calling Protocol, which was also used to derive the final dataset^46^. To improve the genotype calling for rare variants zCall with a threshold of 8 was applied^47^. Individuals with low call rate, discordant sex information (F value between 0.2 and 0.8), related or duplicated individuals (IBD > 0.185) and individuals with divergent ancestry were excluded from further analysis.

#### KORA F4

DNA was extracted from 9 ml EDTA-blood as described elsewhere^48^. Genotyping was done using the Illumina HumanExome v1.0 Bead Array, calling was carried out according to the CHARGE Best Practices and Joint Calling Protocol^46^.

### Metabolite measurements in EPIC-Potsdam and KORA F4

Metabolite quantification of both studies, EPIC-Potsdam and KORA F4, was performed in the Genome Analysis Center at the Helmholtz Zentrum München. All samples have been measured using the Absolute*IDQ* ^TM^ p150 Kit (Biocrates Life Sciences AG, Innsbruck, Austria) in combination with flow injection analysis-tandem mass spectrometry (FIA-MS/MS). Serum samples of 10 µL serum were used to quantify 163 simultaneously, including 41 acylcarnitines (Cx:y), 14 amino acids, hexose (sum of six-carbon monosaccharides without distinction of isomers), 92 glycerophospholipids (lyso-, diacyl-, and acyl-alkylphosphatidylcholines), and 15 sphingomyelins. The method for sample preparation and measurement as well as the metabolite denomination was previously described in full detail^49^. All samples of EPIC-Potsdam have been processed in one batch^50^. The KORA F4 samples have been measured in three batches of approximately 1000 samples at three different time points with a recalibration of the equipment in between^7, 51^; therefore, all analyses calculated in KORA were further adjusted for batch effects. Metabolites showed an overall good reliability over a 4-month period, with a median intraclass correlation coefficient of 0.57^50^. To ensure valid measurements, metabolites below the limit of detection (n = 30) and those with very high analytical variance (n = 6) were excluded, resulting in 127 metabolites^50^. For the present analysis 34 diabetes-associated metabolites and 2 metabolic factors were selected^12^. Previous studies could show that the usage of metabolite ratios compared to single metabolites provides higher statistical power to detect significant associations with genetic variants^52^. Therefore, we selected all metabolite ratios out of the 34 diabetes-associated metabolites which were connected via one edge^17^ based on a metabolite network within EPIC-Potsdam build by Gaussian graphical modeling (Fig. 1)^53^. We included all single metabolites and 2 metabolites factors which were independently associated with type 2 diabetes (Fig. 1)^12^. Therefore, our analysis (Fig. 1) included 106 outcomes referred to as diabetes-related metabolite traits consisting of 90 metabolite ratios, 14 single metabolites and 2 metabolite factors = (list of metabolite traits in Supplementary Table S16).

### Assessment of type 2 diabetes in EPIC-Potsdam

Systematic information sources for incident cases were self-reports of a type 2 diabetes diagnosis, type 2 diabetes-relevant medication, and dietary treatment due to type 2 diabetes during follow-up. Furthermore, we obtained additional information from death certificates or from random sources, such as the tumor centers, physicians, or clinics that provided assessments from other diagnoses. Although self-reports of type 2 diabetes were generally reliable, by including other sources of information, we even improved the completeness of case ascertainment. Once a participant was identified as a potential case, disease status was further verified by sending a standard inquiry form to the treating physician. Only physician- verified cases with a diagnosis of type 2 diabetes (International Classification of Diseases, 10^th^ revision code: E11) and a diagnosis date after the baseline examination were considered confirmed incident cases of type 2 diabetes.

### Statistical analysis

All data analyses were performed by using the software packages Statistical Analysis System (SAS) Enterprise Guide 6.1 with SAS version 9.4 (SAS Institute Inc., Cary, NC, USA), PLINK v1.07^54^ and R (version 3.1.0 (2014-04-10)).

#### Candidate analysis of known mGWAS hits

We selected 19 candidate SNPs that showed associations with diabetes associated metabolites in previously published mGWAS of metabolic traits (Fig. 1). Associations of the selected candidate SNPs (list in Supplementary Table S15)^7, 8^ with metabolite traits were investigated within the sub-cohort via linear regression models (additive genetic model; per copy of the minor allele) adjusted for age and sex using SAS version 9.4. Metabolite traits (except metabolite factors) were natural log-transformed to normalize the right-skewed distributions. After ln-transformation metabolite outliers of >4SD from the mean were excluded and metabolite traits were standardized (mean = 0; SD = 1). Correction for multiple testing was done by the Bonferroni method [significance threshold: 0.05/(19 × 61 independent metabolite traits) = 4.31 × 10^−5^]. The effective number of independent metabolite traits of 61 out of 106 was determined by using equation 5 of Li and Ji 2005^55^ which is included in the matSpD R script available onlin^56, 57^.

#### Exploratory analysis

Exploratory single variant association analysis was performed in PLINK v1.07^54^ and included all exome chip variants with MAF ≥ 0.005 (n~42,000 markers) as exposure and 106 metabolite traits defined above as outcome (Fig. 1). We considered a genomic control (GC) corrected p-value as exome chip-wide significant at *P* < 1.95 × 10^−8^ [ = 0.05/(42,000 polymorphic variants × 61 independent metabolite traits)]^55^. Suggestive significance threshold was defined as P < 1.64 × 10^−7^ [ = (1.00 × 10^−5^/61)]. Transformation of metabolite traits and outlier removal was identical to the replication analysis of previously identified mGWAS hits. We assumed an additive genetic model (per copy of the allele on the forward strand), adjusted for age at recruitment and sex. Identified SNPs with at least suggestive significance (P < 1.64 × 10^−7^) were replicated within the KORA study by applying the same linear models which were further adjusted for batch effects. Results for single variant analysis from EPIC-Potsdam and KORA F4 were pooled by meta-analysis using a random effects model with the R package *meta* ^58^ and significance was defined by correcting for eleven test (P < 4.55 × 10^−3^). Analyses for the top associated metabolite traits were performed to check for independent signals at each locus [*Y* = *β1 SNP*_*1*_ + *β2 SNP*_*2*_ (+*β3 SNP*_*3*_) + *β4 age* + *β5 sex* + *n*]. Independently associated SNPs were selected for the diabetes analysis.

Additionally, we performed an exploratory interaction analysis for sex by modeling linear regression models adjusted for age and sex and including an interaction term (*Y* = *β1 SNP* + *β2 age* + *β3 sex* + *β4* (*sex* × *SNP*) + *n*). Interaction terms with P < 1.64 × 10^−7^ were considered as suggestively significant and attempted to replicate in KORA F4. Gene-based analyses were performed with the R package *SKAT* ^59^. We applied the sequence kernel association test (SKAT-C) and burden-C test with the default options using “SKAT_CommonRare” function^60^ to test for associations between common and rare genetic variants in a gene and 106 metabolite traits (Fig. 1). Models were adjusted for age and sex. Correction for multiple testing was done with the Bonferroni method [suggestive significance threshold: 0.05/(number of genes with >1 variants (ranging from 7243 to 7332)) = 6.8 × 10^−6^ to 6.9 × 10^−6^]. Additional analyses considering only variants annotated as potentially damaging (based on the annotation list from the CHARGE consortium: column “sc_damaging”) were conducted. Genes showing suggestive significance in EPIC-Potsdam were replicated in KORA F4 considering p < 0.05 as replicated. Within genes including more than two variants (*GFRAL*, *TFRC* and *OR51Q1*), we removed the top variant(s) showing the lowest p-values in pooled single variant analyses from the gene-based analyses. By repeating the SKAT and burden-C test we determined the impact of the top variant(s) on the gene-based statistical significance. Variants were mapped to Ensembl annotation version 84 (GRCh37)^26^ and files provided by the CHARGE consortium^46^ were used for gene annotation.

#### Association with type 2 diabetes

We evaluated all replicated previously known and all novel exploratory identified genetic variants (n = 16) with regard to their association with type 2 diabetes risk within EPIC-Potsdam (Fig. 1). Associations between genetic variants and diabetes risk were evaluated in SAS version 9.4 using Cox regression modified for the case-cohort design according to the Prentice method^61^. Age was used as the underlying time scale in all Cox models, with entry time defined as the participant’s age at recruitment and exit time defined as the age at the end of follow-up based on the date of diagnosis, death, or return of the last follow-up questionnaire. The analysis was stratified for age at the baseline examination in one-year intervals. Cox models were further adjusted for sex and waist circumference.

Associations between the investigated SNPs and risk of type 2 diabetes as well as BMI and fasting glucose were looked up in the AMP-T2D Program^14^. This database provides information on genetic variants and their diabetes-association in consortia for type 2 diabetes using exome sequencing, exome arrays for low-frequency variants and arrays for common variants^14^. Additionally, GWAS meta-analysis results for 24 other traits are available^14^.

Correction for multiple testing was applied by using false discovery rate (FDR) according to Benjamini and Hochberg^62^. We defined the expected direction for the SNP-diabetes association based on the sign of the product between the SNP-metabolite trait association (e.g. rs3204953 (*REV3L*) → tyrosine/methionine) and the metabolite trait-type 2 diabetes association (e.g. tyrosine/methionine → type 2 diabetes risk). For the latter we selected either the metabolite trait showing the lowest p-value with the SNP or the one of the SNP-associated metabolite traits indicating significant association with type 2 diabetes risk adjusted for age and sex in EPIC-Potsdam. We tested for directional consistency between expected and observed direction by applying a binomial test in R.

### Assessment of functionality of genetic variants

To estimate the relative pathogenicity of identified genetic variants, we used Combined Annotation Dependent Depletion (CADD) v1.3^25^. This method integrates various annotations into a single measure (C score) for each genetic variant. The scaled C score ranges from 1 to 99 and ranks each variant in the genome relative to all possible 8.6 billion substitutions in the human reference genome based on the formula −10 × log_10_ (rank/total number of substitutions)^25^.

### Bioinformatics annotation of genes at metabolite-associated loci

All identified SNPs were systematically linked to genes using a flanking region of at least 500 kb. A “GWAS to pathway” workflow method^63^ was then used to link these genes to potential pathways within the KEGG database^64^. We used the Taverna Workbench Bioinformatics 2.5^65^ software to run the workflow.

## Electronic supplementary material

Supplementary PDF File
