# Supplementary material for: Genetic variants including markers from the exome chip and metabolite traits of type 2 diabetes
Source: Sci Rep. 2017 Jul 20;7:6037. doi: 10.1038/s41598-017-06158-3 (PMC5519666; doi:10.1038/s41598-017-06158-3)
Supplement: Supplementary file 1 — Supplementary PDF File [file 41598_2017_6158_MOESM1_ESM.pdf]

Genetic variants including markers from the exome chip and metabolite traits of type 2 diabetes

Susanne Jäger[1,2], Simone Wahl[2,3,4], Janine Kröger[1,2], Sapna Sharma[2,3,4], Per Hoffmann[5,6,7], Anna Floegel[8], Tobias Pischon[9,10,11], Cornelia Prehn[12], Jerzy Adamski[2,12,13], Martina Müller-Nurasyid[14,15,16], Melanie Waldenberger[3,4], Konstantin Strauch[14,17], Annette Peters[2,3,16], Christian Gieger[3,4], Karsten Suhre[18], Harald Grallert [2,3,4], Heiner Boeing[8], Matthias B. Schulze[1,2], Karina Meidtner[1,2]

[1] Department of Molecular Epidemiology, German Institute of Human Nutrition Potsdam-Rehbruecke, Nuthetal, Germany

[2] German Center for Diabetes Research (DZD), Neuherberg, Germany

[3] Institute of Epidemiology II, Helmholtz Zentrum München, German Research Center for Environmental Health, Neuherberg, Germany

[4] Research Unit of Molecular Epidemiology, Helmholtz Zentrum München, German Research Center for Environmental Health, Neuherberg, Germany

[5] Division of Medical Genetics, Department of Biomedicine, University of Basel, Basel, Switzerland

[6] Department of Genomics, Life and Brain Center, Bonn, Germany

[7] Institute of Human Genetics, University of Bonn, Bonn, Germany

[8] Department of Epidemiology, German Institute of Human Nutrition Potsdam-Rehbruecke, Nuthetal, Germany

[9] Molecular Epidemiology Group Max Delbrueck Center for Molecular Medicine in the Helmholtz Association (MDC) Berlin-Buch Germany

[10] Charité – Universitätsmedizin Berlin, Berlin, Germany

[11] DZHK (German Centre for Cardiovascular Research), partner site Berlin, Berlin, Germany

[12] Genome Analysis Center, Institute of Experimental Genetics, Helmholtz Zentrum München, German Research Center for Environmental Health, Neuherberg, Germany

[13] Chair of Experimental Genetics, Center of Life and Food Sciences Weihenstephan, Technische Universität München, Freising-Weihenstephan, Germany

[14] Institute of Genetic Epidemiology, Helmholtz Zentrum München, German Research Center for Environmental Health, Neuherberg, Germany

[15] Department of Medicine I, University Hospital Grosshadern, Ludwig-Maximilians-Universität, Munich, Germany

[16] DZHK (German Centre for Cardiovascular Research), partner site Munich Heart Alliance, Munich, Germany

[17] Institute of Medical Informatics, Biometry and Epidemiology, Chair of Genetic Epidemiology, Ludwig-Maximilians-Universität, Munich, Germany

[18] Department of Physiology and Biophysics, Weill Cornell Medical College in Qatar, Qatar Foundation-Education City, Doha, Qatar

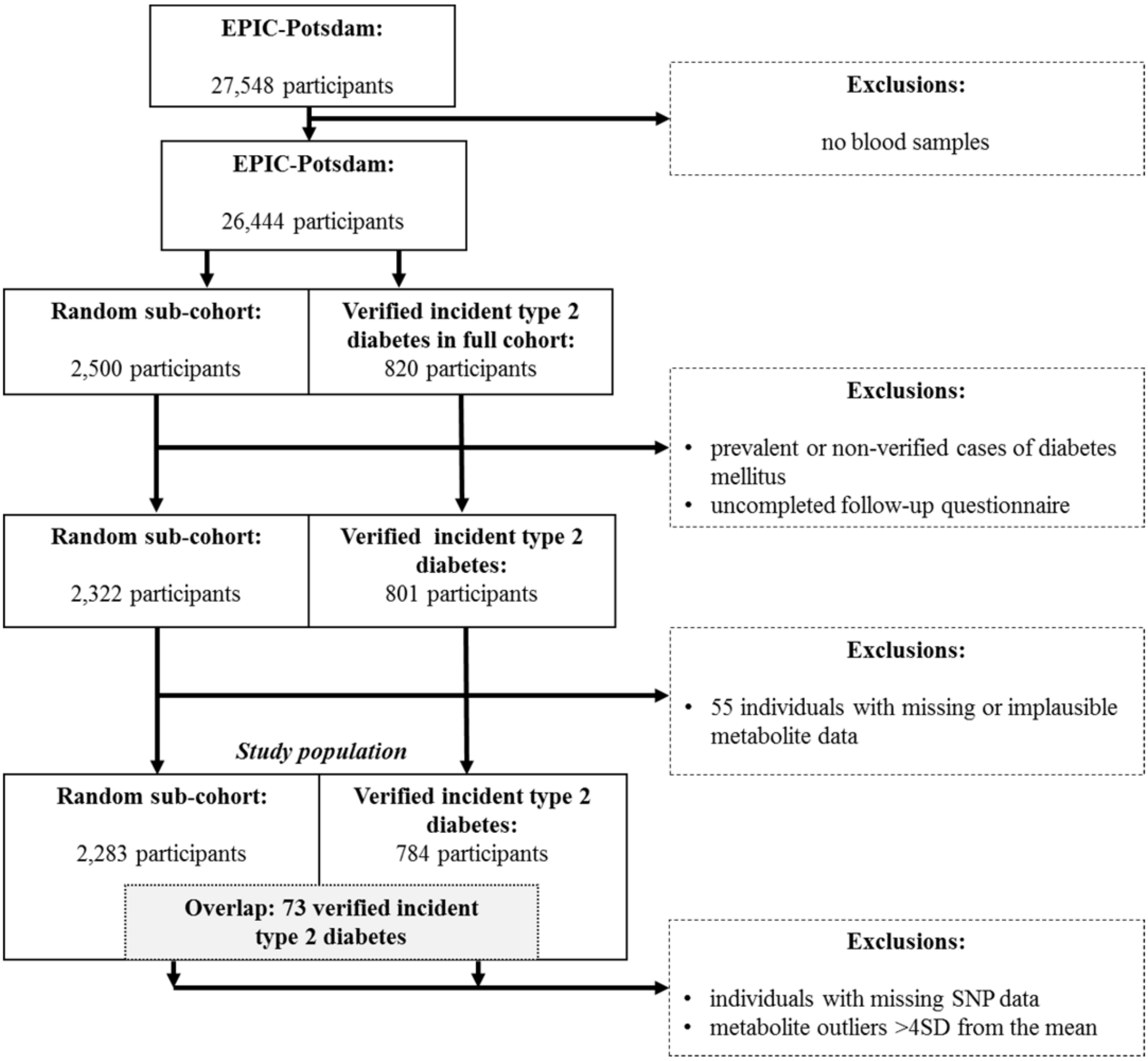

Supplementary Fig. S1. Design of the nested case-cohort study in EPIC-Potsdam with randomly drawn sub-cohort and incident cases of type 2 diabetes

Supplementary Table S1. Baseline characteristics in EPIC-Potsdam and KORA F4

	EPIC-Potsdam	KORA F4
N (%women)	2283 (61.8)	2818 (47.6)
Age at baseline in years	49 (15)	55 (22)
BMI in kg/m²	25.5 (5.18)	.
Waist circumference in cm	85.0 (18.8)	.
Hexose in µmol/L	4556 (886)	4962 (781)
Glycine in µmol/L	241 (85.0)	295 (93.0)
Isoleucine + Leucine in µmol/L	200 (69.0)	206 (59.0)
Phenylalanine in µmol/L	54.9 (13.3)	61.0 (13.5)
Tryptophan in µmol/L	80.0 (15.1)	82.6 (12.6)
Tyrosine in µmol/L	78.6 (27.4)	83.2 (23.2)
Valine in µmol/L	286 (83.0)	270 (83.0)
C3 in µmol/L	0.36 (0.18)	0.37 (0.15)
PC ae C32:1 in µmol/L	2.81 (0.79)	2.81 (0.87)
PC ae C32:2 in µmol/L	0.71 (0.22)	0.74 (0.24)
PC ae C34:2 in µmol/L	13.0 (4.60)	12.4 (4.50)
PC ae C34:3 in µmol/L	8.21 (3.11)	8.17 (3.12)
PC ae C36:2 in µmol/L	16.8 (5.60)	14.8 (5.30)
PC ae C36:3 in µmol/L	9.36 (2.93)	8.47 (2.80)
PC ae C40:5 in µmol/L	3.91 (1.01)	3.52 (0.88)
PC ae C40:6 in µmol/L	5.36 (1.81)	4.90 (1.70)
PC ae C42:3 in µmol/L	0.84 (0.25)	0.85 (0.27)
PC ae C42:4 in µmol/L	0.92 (0.29)	0.99 (0.33)
PC ae C42:5 in µmol/L	2.30 (0.67)	2.30 (0.64)
PC ae C44:4 in µmol/L	0.37 (0.13)	0.42 (0.14)
PC ae C44:5 in µmol/L	1.72 (0.60)	2.06 (0.71)
PC ae C44:6 in µmol/L	1.14 (0.40)	1.34 (0.48)
PC aa C32:1 in µmol/L	14.7 (10.0)	19.0 (11.8)
PC aa C36:1 in µmol/L	55.4 (17.7)	51.8 (16.5)
PC aa C36:3 in µmol/L	149 (43.0)	147 (42.0)
PC aa C38:3 in µmol/L	53.5 (18.2)	52.2 (17.7)
PC aa C40:4 in µmol/L	3.83 (1.34)	3.94 (1.40)

PC aa C40:5 in µmol/L	10.9 (4.32)	11.0 (3.80)
PC aa C42:0 in µmol/L	0.58 (0.22)	0.57 (0.22)
PC aa C42:1 in µmol/L	0.29 (0.11)	0.29 (0.10)
SM C16:1 in µmol/L	17.3 (4.70)	15.6 (4.60)
SM (OH) C22:2 in µmol/L	12.0 (4.31)	11.2 (3.83)
LysoPC a C17:0 in µmol/L	1.94 (0.77)	1.68 (0.69)
LysoPC a C18:2 in µmol/L	33.2 (17.0)	25.9 (12.1)

Age, BMI, waist circumference and 34 diabetes-associated metabolites are depicted as medians (interquartile range)

Supplementary Table S2. Replicated genetic variants associated with diabetes-associated metabolite traits in EPIC-Potsdam

SNP	Reported Locus	Chr	Most severe consequence *	Scaled CADD score [1]	Minor allele	MAF	Metabolite trait	N	β (95% CI) [a]	p-value [b]	Adj. R^2 in %
rs541503 [c]	*PHGDH*	1	intron variant	4.82	C	37.5	Glycine/Serine	2204	0.1677 (0.1071 -0.2283)	6.31E-08	2.87
rs715 [d]	*CPS1*	2	3 prime UTR variant	3.20	C	30.1	Glycine	2196	0.5870 (0.5277 -0.6464)	1.81E-77	16.1
							Glycine/Serine	2197	0.4895 (0.4286 -0.5504)	3.77E-53	11.7
							Serine/Phenylalanine	2197	0.1763 (0.1137 -0.2388)	3.65E-08	6.76
rs12641551 [c]	*ACSL1*	4	intron variant	3.93	G	31.9	PC ae C44:5/ PC ae C42:5	2202	0.2533 (0.1915 -0.3150)	1.36E-15	3.47
							PC ae C44:6/ PC aa C42:1	2203	0.1347 (0.0728 -0.1966)	2.04E-05	2.87
rs272893 [c]	*SLC22A4, OCTN1*	5	Ile306Thr	12.57	A	38.4	PC ae C44:5/ PC ae C42:5	2202	0.1406 (0.0812 -0.2000)	3.70E-06	1.59
rs9393903 [c]	*ELOVL2*	6	intron variant	2.19	A	24.5	PC aa C40:4/ PC aa C42:6	2240	0.1582 (0.0915 -0.2249)	3.52E-06	3.11
							PC aa C40:5/ PC aa C42:5	2240	0.2276 (0.1624 -0.2928)	9.74E-12	7.50
							PC aa C40:5/ PC aa C40:6	2241	0.1499 (0.0836 -0.2161)	9.59E-06	4.43
rs603424 [c]	*SCD*	10	intron variant	6.86	A	18.7	PC ae C42:5/ PC ae C40:4	2195	-0.1632 (-0.2382--0.0883)	2.04E-05	0.99
							SM (OH) C22:2/ SM C18:1	2194	-0.1828 (-0.2574--0.1081)	1.71E-06	1.74
							SM C16:1/ SM C18:1	2190	-0.1565 (-0.2305--0.0826)	3.45E-05	3.79
							PC aa C36:3/	2192	0.1745	4.32E-06	2.89

							PC aa C34:3		(0.1002 -0.2488)		
							PC aa C32:1/ PC aa C34:1	2193	-0.1734 (-0.2479--0.0988)	5.36E-06	2.20
							SM (OH) C22:2/ SM C18:0	2193	-0.1529 (-0.2251--0.0807)	3.41E-05	8.18
rs174547 [c]	*FADS1*	11	intron variant	6.23	G	33.5	PC ae C40:6	2204	-0.1288 (-0.1880--0.0695)	2.09E-05	7.12
							PC ae C42:5	2203	-0.2469 (-0.3057--0.1881)	3.00E-16	8.52
							PC ae C44:5	2204	-0.2031 (-0.2628--0.1433)	3.41E-11	5.38
							PC aa C40:5	2203	-0.2194 (-0.2786--0.1601)	5.28E-13	7.09
							PC ae C34:1/ PC ae C32:1	2200	0.1593 (0.0987 -0.2199)	2.75E-07	2.91
							PC ae C34:2/ PC ae C38:5	2204	0.4321 (0.3770 -0.4872)	8.09E-51	19.7
							PC ae C34:2/ PC ae C36:4	2203	0.4302 (0.3749 -0.4855)	4.60E-50	19.1
							PC ae C34:2/ PC ae C36:3	2202	-0.1384 (-0.1990--0.0778)	7.92E-06	2.79
							PC aa C36:3/ PC aa C36:5	2201	0.3573 (0.2991 -0.4155)	2.39E-32	10.3
							PC ae C36:3/ PC ae C38:5	2204	0.5707 (0.5178 -0.6237)	1.91E-90	25.8
							PC ae C36:3/ PC ae C34:3	2204	0.2875 (0.2275 -0.3474)	1.28E-20	4.81
							PC ae C34:3/ PC ae C36:5	2204	0.3832 (0.3275 -0.4389)	6.63E-40	17.8
							PC ae C40:6/ PC ae C38:4	2202	0.1954 (0.1359 -0.2549)	1.47E-10	6.31
							PC ae C42:4/ PC ae C40:4	2202	0.2263 (0.1660 -0.2865)	2.53E-13	3.91

PC ae C42:4/ PC ae C44:4	2200	-0.2573 (-0.3178--0.1968)	1.28E-16	3.22
PC ae C44:4/ PC ae C44:5	2201	0.4018 (0.3429 -0.4606)	2.39E-39	8.38
PC ae C44:6/ PC aa C42:0	2204	-0.1787 (-0.2386--0.1188)	5.69E-09	4.98
PC aa C42:0/ PC ae C42:5	2204	0.1273 (0.0674 -0.1872)	3.17E-05	5.08
PC ae C40:5/ PC ae C36:0	2203	-0.1381 (-0.1988--0.0774)	8.53E-06	2.46
PC ae C42:3/ PC ae C42:2	2202	0.1701 (0.1113 -0.2289)	1.59E-08	8.52
PC ae C42:3/ PC ae C40:1	2202	0.2522 (0.1941 -0.3103)	2.93E-17	10.8
SM (OH) C22:2/ PC ae C38:2	2198	-0.1699 (-0.2300--0.1099)	3.17E-08	4.89
PC aa C40:5/ PC aa C36:5	2203	0.1419 (0.0809 -0.2029)	5.31E-06	1.51
PC aa C40:5/ PC aa C36:3	2204	-0.3824 (-0.4389--0.3259)	9.65E-39	15.5
PC aa C40:5/ PC aa C38:5	2203	0.1624 (0.1022 -0.2226)	1.33E-07	4.16
PC aa C36:3/ PC aa C36:4	2201	0.7445 (0.6919 -0.7971)	1.71E-145	26.7
PC aa C36:3/ PC aa C38:4	2204	0.7213 (0.6684 -0.7742)	1.31E-136	25.9
PC aa C36:3/ PC aa C38:3	2202	0.1901 (0.1313 -0.2490)	2.83E-10	8.42
PC aa C36:3/ PC aa C38:5	2201	0.5330 (0.4772 -0.5888)	8.70E-73	17.7
PC aa C38:3/ PC aa C36:5	2201	0.2954 (0.2356 -0.3553)	9.90E-22	5.23
PC aa C38:3/ PC aa C38:4	2204	0.7252 (0.6728 -0.7776)	4.85E-140	27.2

							PC aa C38:3/ PC aa C36:4	2204	0.4537 (0.3972 -0.5103)	6.76E-53	15.2
							PC aa C38:3/ PC aa C38:5	2201	0.4306 (0.3721 -0.4892)	4.08E-45	9.30
							PC ae C36:2/ PC ae C36:1	2203	0.1981 (0.1387 -0.2575)	7.52E-11	6.66
							PC ae C36:2/ LysoPC a C17:0	2202	0.1847 (0.1262 -0.2432)	7.01E-10	9.52
							LysoPC a C18:2/ LysoPC a C20:4	2204	0.4978 (0.4401 -0.5556)	2.28E-60	11.7
rs1718306 [d]	*PAH*	12	intron variant	0.72	T	39.9	Phenylalanine/ Arginine	2211	0.1526 (0.0922 -0.2131)	7.92E-07	1.89
rs7156144 [c]	*PLEKHH1*	14	intron variant	0.38	A	42.5	PC ae C34:1/ PC ae C32:1	2174	0.2608 (0.2004 -0.3212)	4.52E-17	4.80
							PC ae C34:3/ PC ae C36:5	2178	0.1955 (0.1377 -0.2532)	3.96E-11	12.7
rs11158519 [c]	*SYNE2*	14	intron variant	5.24	A	13.5	SM (OH) C22:2/ SM(OH)C22:1	2209	0.2092 (0.1300 -0.2885)	2.47E-07	13.4
							SM (OH) C22:2/ SM(OH)C14:1	2210	-0.3690 (-0.4511--0.2868)	2.50E-18	6.97
							SM C16:1/ PC aa C28:1	2210	-0.3822 (-0.4658--0.2987)	5.93E-19	3.81
rs364585 [c]	*SPTLC3*	20	intergenic variant	0.77	A	38.1	SM (OH) C22:2/ SM C16:1	2201	0.1552 (0.0979 -0.2125)	1.22E-07	6.56
							SM C16:1/ SM C18:0	2202	-0.1866 (-0.2451--0.1281)	4.79E-10	2.66
							SM C16:1/ PC aa C28:1	2203	-0.1441 (-0.2030--0.0853)	1.68E-06	1.36

CADD, Combined Annotation Dependent Depletion; MAF, minor allele frequency

[a] metabolite traits (μmol/L) were ln-transformed, outliers (>4 SD) were removed and metabolite traits were standardized, models are adjusted for age and sex;

[b] significance threshold: 0.05/(19 × 61 Outcomes) = 4.31E-05; [c] reported in study from Illig et al. 2010[2]; [d] reported in study from Shin et al. 2014 [3];

*Ensembl annotation version 84 (GRCh37)

Supplementary Table S3. Replicated genetic variants and diabetes-associated metabolite traits with significant sex-interaction

SNP	Locus	Chr	Minor allele	MAF in %	Metabolite trait	Women				Men				p-value (interaction) [b]
						N	β (95% CI)[a]	P-value	Adj. R^2 in %	N	β (95% CI)[a]	P-value	Adj. R^2 in %	
rs715[c]	*CPS1*	2	C	30.1	Glycine	1361	0.6920 (0.6181 - 0.7659)	1.77E-67	20.8	835	0.4016 (0.3015 - 0.5016)	1.06E-14	6.94	4.02E-14
					Glycine/Serine	1361	0.6106 (0.5358 - 0.6854)	5.81E-53	18.8	836	0.2623 (0.1601 - 0.3646)	5.86E-07	2.73	6.36E-12

MAF, minor allele frequency

[a] metabolite traits (µmol/L) were ln-transformed, outliers (>4 SD) were removed and metabolite traits were standardized, models are adjusted for age;
[b] significance threshold for interaction of SNP with sex: 0.05/(19 SNPs × 61 Outcomes) = 4.31E-05; [c] reported in study from Shin et al. 2014 [3];

Supplementary Table S4. Exploratory identified exome chip variants and diabetes-associated metabolite traits with suggestive significance within EPIC-Potsdam

Metabolite trait	Exome chip name	SNP	Locus	Chr	Coded allele on forward strand [a]	CAF in %	N	p-value (GC) [b]	Beta [c]	SE	Most severe consequence *	Scaled CADD score [1]
PC aa C36:3/ PC aa C36:4	exm-rs174550	rs174550	*FADS1*	11	T	66.5	2201	1.16E-146	-0.7470	0.0268	5 prime UTR variant	3.57
PC aa C38:3/ PC aa C38:4	exm-rs174550	rs174550	*FADS1*	11	T	66.5	2204	2.09E-140	-0.7259	0.0267	5 prime UTR variant	3.57
PC aa C36:3/ PC aa C38:4	exm-rs174550	rs174550	*FADS1*	11	T	66.5	2204	4.07E-137	-0.7224	0.0270	5 prime UTR variant	3.57
PC ae C36:3/ PC ae C38:5	exm-rs174550	rs174550	*FADS1*	11	T	66.5	2204	1.13E-88	-0.5712	0.0270	5 prime UTR variant	3.57
PC aa C36:3/ PC aa C38:5	exm-rs174550	rs174550	*FADS1*	11	T	66.5	2201	3.06E-73	-0.5346	0.0284	5 prime UTR variant	3.57
LysoPC a C18:2/ LysoPC a C20:4	exm-rs174546	rs174546	*FADS1*	11	C	66.6	2204	9.16E-60	-0.4984	0.0294	3 prime UTR variant	12.1
PC aa C38:3/ PC aa C36:4	exm-rs174550	rs174550	*FADS1*	11	T	66.5	2204	1.02E-52	-0.4546	0.0288	5 prime UTR variant	3.57
PC ae C34:2/ PC ae C36:4	exm-rs174546	rs174546	*FADS1*	11	C	66.6	2203	1.15E-49	-0.4303	0.0282	3 prime UTR variant	12.1
PC ae C34:2/ PC ae C38:5	exm-rs174546	rs174546	*FADS1*	11	C	66.6	2204	5.17E-49	-0.4323	0.0281	3 prime UTR variant	12.1
PC aa C38:3/ PC aa C38:5	exm-rs174550	rs174550	*FADS1*	11	T	66.5	2201	3.25E-45	-0.4310	0.0299	5 prime UTR variant	3.57
PC ae C44:4/ PC ae C44:5	exm-rs174550	rs174550	*FADS1*	11	T	66.5	2201	2.37E-39	-0.4024	0.0300	5 prime UTR variant	3.57
PC ae C34:3/ PC ae C36:5	exm-rs174546	rs174546	*FADS1*	11	C	66.6	2204	5.05E-39	-0.3841	0.0284	3 prime UTR variant	12.1
PC aa C40:5/ PC aa C36:3	exm-rs174550	rs174550	*FADS1*	11	T	66.5	2204	1.41E-38	0.3831	0.0288	5 prime UTR variant	3.57
SM C16:1/ PC aa C28:1	exm-rs7157785	rs7157785	*SGPP1*	14	G	83.6	2203	1.45E-35	0.4924	0.0389	Regulatory region variant	1.91

PC aa C36:3/ PC aa C36:5	exm-rs174550	rs174550	*FADS1*	11	T	66.5	2201	4.16E-32	-0.3582	0.0297	5 prime UTR variant	3.57
SM(OH)C22:2/ SM(OH)C14:1	exm-rs7157785	rs7157785	*SGPP1*	14	G	83.6	2202	2.79E-27	0.4234	0.0385	Regulatory region variant	1.91
PC aa C38:3/ PC aa C36:5	exm-rs174550	rs174550	*FADS1*	11	T	66.5	2201	3.44E-21	-0.2956	0.0305	5 prime UTR variant	3.57
PC ae C36:3/ PC ae C34:3	exm-rs174583	rs174583	*FADS2*	11	C	66.1	2204	2.39E-20	-0.2896	0.0306	intron variant	13.8
Glycine	exm2269212	rs4672596	*CPS1*	2	T	39.2	2203	1.73E-17	-0.2616	0.0303	intergenic	3.86
PC ae C42:3/ PC ae C40:1	exm-rs174550	rs174550	*FADS1*	11	T	66.5	2202	4.26E-17	-0.2536	0.0296	5 prime UTR variant	3.57
PC ae C42:4/ PC ae C44:4	exm-rs174546	rs174546	*FADS1*	11	C	66.6	2200	8.67E-17	0.2586	0.0308	3 prime UTR variant	12.1
PC ae C44:5/ PC ae C42:5	exm2269890	rs12641551	*ACSL1*	4	C	32.0	2202	1.21E-15	0.2537	0.0315	intron variant	3.93
PC ae C42:5	exm-rs174547	rs174547	*FADS1*	11	T	66.5	2203	1.21E-15	0.2471	0.0300	intron variant	3.57
SM(OH)C22:2/ SM(OH)C22:1	exm-rs7157785	rs7157785	*SGPP1*	14	G	83.6	2202	6.00E-15	-0.2971	0.0372	Regulatory region variant	1.91
SM(OH)C22:2/ SM(OH)C14:1	exm1106800	rs17751301	*SYNE2*	14	C	92.9	2202	1.22E-14	0.4382	0.0563	Arg1393Trp	10.1
SM C16:1/ PC aa C28:1	exm1106800	rs17751301	*SYNE2*	14	C	92.9	2203	2.35E-14	0.4401	0.0573	Arg1393Trp	10.1
PC ae C42:4/ PC ae C40:4	exm-rs174550	rs174550	*FADS1*	11	T	66.5	2202	3.60E-13	-0.2270	0.0307	5 prime UTR variant	3.57
Gly/Ser	exm2269212	rs4672596	*CPS1*	2	T	39.2	2204	4.58E-13	-0.2238	0.0305	intergenic	3.86
PC aa 40:5	exm-rs174547	rs174547	*FADS1*	11	T	66.5	2203	5.16E-13	0.2197	0.0302	intron variant	3.57
Tyr/Met	exm572044	rs3204953	*REV3L*	6	C	85.2	2201	9.80E-12	-0.2841	0.0412	Val3064Ile	32.0
PC aa C36:3/ PC aa C38:3	exm-rs174570	rs174570	*FADS2*	11	C	85.7	2202	3.63E-11	-0.2739	0.0412	intron variant	3.93
PC ae C44:5	exm-rs174547	rs174547	*FADS1*	11	T	66.5	2204	1.11E-10	0.2031	0.0305	intron variant	3.57

PC ae C40:6/ PC ae C38:4	exm-rs174546	rs174546	*FADS1*	11	C	66.5	2202	1.28E-10	-0.1980	0.0303	3 prime UTR variant	12.1
PC ae C36:2/ PC ae C36:1	exm-rs174547	rs174547	*FADS1*	11	T	66.5	2203	1.36E-10	-0.1973	0.0303	intron variant	3.57
PC ae C36:2/ LysoPC a C17:0	exm-rs102275	rs102275	*C11orf10*	11	T	65.9	2202	2.90E-10	-0.1889	0.0297	Non coding transcript exon variant	6.59
SM C16:1/ PC aa C28:1	exm1107280	rs12881815	*SYNE2*	14	G	95.2	2203	3.25E-10	0.4347	0.0688	Glu4913Lys	25.2
SM(OH)C22:2/ SM C24:0	exm-rs7157785	rs7157785	*SGPP1*	14	G	83.6	2203	5.46E-10	-0.2221	0.0353	Regulatory region variant	1.91
SM C16:1/ SM C18:0	exm-rs364585	rs364585	*SPTLC3*	20	A	38.1	2202	6.78E-10	-0.1854	0.0298	intergenic	0.77
PC aa C38:3/ PC ae C36:2	exm-rs174570	rs174570	*FADS2*	11	C	85.7	2202	7.13E-10	0.2589	0.0415	intron variant	3.93
SM C16:1/ SM C18:0	exm-rs680379	rs680379	*SPTLC3*	20	A	38.1	2202	1.12E-09	-0.1829	0.0298	intergenic	0.66
PC ae C42:3/ PC ae C42:2	exm-rs102275	rs102275	*C11orf10*	11	T	65.9	2202	5.12E-09	-0.1753	0.0299	Non coding transcript exon variant	6.59
PC aa C36:3/ PC aa C34:3	exm1219342	rs1136001	*NTAN1*	16	G	67.0	2201	5.58E-09	0.1835	0.0314	His283Asn	0.81
PC aa C36:3/ PC aa C34:3	exm1219341	rs1135999	*NTAN1*	16	A	67.0	2201	5.58E-09	0.1835	0.0314	Ser287Pro	13.3
PC aa C36:3/ PC aa C34:3	exm-rs7200543	rs7200543	*PDXDC1*	16	A	67.0	2201	5.58E-09	0.1835	0.0314	Synonymous variant	5.10
PC ae C44:6/ PC aa C42:0	exm-rs174546	rs174546	*FADS1*	11	C	66.6	2204	7.79E-09	0.1799	0.0305	3 prime UTR variant	12.1
PC aa C40:5/ PC aa C38:5	exm-rs499974	rs499974	*MOGAT2*	11	C	81.2	2203	2.25E-08	-0.2143	0.0379	Downstream gene variant	8.66
PC aa C36:1/ PC aa C34:1	exm858668	rs4751995	*PNLIPRP2*	10	A	47.6	2201	2.90E-08	-0.1692	0.0297	intron variant/ splice region variant	10.4
SM(OH)C22:2/ PC ae C38:2	exm-rs174546	rs174546	*FADS1*	11	C	66.5	2198	4.29E-08	0.1683	0.0306	3 prime UTR variant	12.1

PC aa C36:1/ PC aa C34:1	exm858674	rs10885997	*PNLIPRP2*	10	A	58.8	2201	5.54E-08	-0.1697	0.0304	Synonymous variant	7.74
SM(OH)C22:2/ SM(OH)C14:1	exm1107280	rs12881815	*SYNE2*	14	G	95.2	2202	6.95E-08	0.3677	0.0678	Glu4913Lys	25.2
PC aa C42:1/ PC aa C42:0	exm83509	rs41282492	*CHIA*	1	A	87.9	2190	1.01E-07	0.2501	0.0463	Asn45Asp	0.01
SM(OH)C22:2/ SM C16:1	exm-rs364585	rs364585	*SPTLC3*	20	A	38.1	2201	1.23E-07	0.1550	0.0292	intergenic	0.77
SM(OH)C22:2/ SM C16:1	exm-rs680379	rs680379	*SPTLC3*	20	A	38.1	2201	1.28E-07	0.1548	0.0292	intergenic	0.66
SM(OH)C22:2/ SM(OH)C22:1	exm1479366	rs7412	*APOE*	19	C	91.4	2202	1.30E-07	-0.2677	0.0498	Arg202Cys	30.0
PC aa C42:1/ PC aa C42:0	exm83510	rs41282494	*CHIA*	1	G	87.8	2200	1.33E-07	0.2465	0.0460	Asp47Asn	26.0
PC aa C40:5/ PC aa C38:5	exm-rs174550	rs174550	*FADS1*	11	T	66.5	2203	1.38E-07	-0.1634	0.0307	5 prime UTR variant	3.57
PC ae C44:6/ PC aa C42:1	exm-rs10790162	rs10790162	*BUD13*	11	A	6.70	2203	1.57E-07	-0.3201	0.0605	intron variant	6.55

CAF, coded allele frequency; CADD, Combined Annotation Dependent Depletion; SE, standard error

[a] gene variants are reported on the forward strand of NCBI build 37; [b] suggestive significance was defined as P < 1.64E-7 = (1E-5/ 61);
[c] metabolite traits (μmol/L) were ln-transformed, outliers (>4 SD) were removed and metabolite traits were standardized, models are adjusted for age and sex;
* Ensembl annotation version 84 (GRCh37)

The *FADS* region is characterized by high linkage disequilibrium (LD); therefore, only the results for the top associated *FADS* variant for each metabolite trait are depicted.

Supplementary Table S5. Identification of independent signals for *CPS1*, *FADS1* and *SGPP1/SYNE2* loci for selected metabolite traits within EPIC-Potsdam

Metabolite trait	Locus	N	SNPs in the model	Coded allele	Beta[a]	SE	P-value[b]	LD between SNPs[c]
Glycine	*CPS1*	2123	**rs715**	C	0.56937	0.03424	<.0001	
			rs4672596	A	-0.02421	0.03224	0.4529	r²=0.2; D‘=0.8
PC aa C36:3/ PC aa C36:4	*FADS1*	2201	**rs174550**	G	2.14218	0.85492	0.0123	
			rs174547	G	-1.39611	0.85505	0.1027	r²=1.0; D‘=1.0
SM C16:1/ PC aa C28:1	*SGPP1/SYNE2*	2137	**rs7157785**	A	-0.52937	0.06265	<.0001	
			rs12881815	A	-0.14242	0.07787	0.0675	r²=0.2; D‘=0.8
			rs11158519	A	0.09414	0.06985	0.1778	r²=0.6; D‘=0.9

LD, linkage disequilibrium; SE, standard error

SNPs selected for the analysis on type 2 diabetes are depicted in bold

[a] metabolites (µmol/L) were ln-transformed , outliers (>4 SD) were removed and z-transformation was applied, analyses were conducted to check for independent signals at each locus [$Y= \beta 1\ SNP_1 + \beta 2\ SNP_2\ (+ \beta 3\ SNP_3) + \beta 4\ age + \beta 5\ sex + n$]; [b] significant SNPs (in bold) were selected for type 2 diabetes analyses; [c] depicted values for LD between respective SNP in that row and selected SNP for type 2 diabetes analysis (in bold)

Supplementary Table S6. Exploratory identified exome chip variants and diabetes-associated metabolite traits with significant sex-interaction within EPIC-Potsdam

			EPIC-Potsdam								KORA F4									
				Women			Men					Women			Men					
Exome chip name (SNP)	Locus (Chr)	Metabolite trait	Coded allele (CAF in %)	N	β (SE)[a]	P-value	N	β (SE)[a]	P-value	P-value[b] (interaction)	Coded allele (CAF in %)	N	β (SE)[a]	P-value	N	β (SE)[a]	P-value	P-value[b] (interaction)	Most severe consequence * (Scaled CADD score[1])	replicated in KORA F4
exm151565 (rs764535)	*TLR5* (1)	LysoPC a C18:2/ LysoPC a C18:1	G (99.0)	1368	-0.166 (0.155)	2.84E-01	834	1.833 (0.3326)	4.79E-08	1.62E-08	G (99.0)	1409	0.222 (0.224)	0.3216	1282	0.012 (0.1672)	0.9428	na	Thr82Ile (1.50)	no
exm1624797 (rs138899368)	*CRLF2* (X)	LysoPC a C18:2/ LysoPC a C18:1	C (98.5)	1368	-0.240 (0.131)	6.69E-02	805	7.429 (1.038)	1.86E-12	1.26E-14	C (97.7)	1408	0.118 (0.105)	0.2621	1283	-0.0173 (0.145)	0.9051	0.4176	Val136Met (.)	no
exm1624797 (rs138899368)	*CRLF2* (X)	PC aa C36:1/ PC aa C34:2	C (98.5)	1368	-0.042 (0.138)	7.58E-01	804	-5.990 (0.950)	4.67E-10	1.31E-09	C (97.7)	1408	-0.046 (0.080)	0.5624	1283	-0.0962 (0.1036)	0.3529	0.6618	Val136Met (.)	no
exm1624797 (rs138899368)	*CRLF2* (X)	PC aa C36:1/ PC aa C36:2	C (98.5)	1367	0.030 (0.132)	8.18E-01	804	-6.279 (1.043)	2.62E-09	2.42E-10	C (97.7)	1408	-0.054 (0.101)	0.5977	1282	-0.1027 (0.1332)	0.4408	0.7799	Val136Met (.)	no

CAF, coded allele frequency; CADD, Combined Annotation Dependent Depletion; SE, standard error

[a] metabolite traits (µmol/L) were ln-transformed, outliers (>4 SD) were removed and metabolite traits were standardized, models are adjusted for age; [b] significance threshold for interaction of SNP with sex: 1E-05/61 Outcomes = 1.64E-07; * Ensembl annotation version 84 (GRCh37)

Supplementary Table S7. Summary of biologic pathway annotations from KEGG database

Chr	Locus	SNP	Entrez gene ID	Annotated gene	set flanking width around the SNP (+/- kb)	KEGG pathway
1	*CHIA*	rs41282494	27159	*CHIA*	500	hsa00520 Amino sugar and nucleotide sugar metabolism
1	*PHGDH* *	rs541503	26227	*PHGDH*	500	hsa00260 Glycine, serine and threonine metabolism
						hsa01100 Metabolic pathways
						hsa01130 Biosynthesis of antibiotics
						hsa01200 Carbon metabolism
						hsa01230 Biosynthesis of amino acids
2	*CPS1* *	rs715	1373	*CPS1*	500	hsa00220 Arginine biosynthesis
						hsa00250 Alanine, aspartate and glutamate metabolism
						hsa00910 Nitrogen metabolism
						hsa01100 Metabolic pathways
						hsa01200 Carbon metabolism
						hsa01230 Biosynthesis of amino acids
2	*CPS1*	rs4672596	29034	*CPS1 intronic transcript 1*	200000	x
			1373	*CPS1*	200000	hsa00220 Arginine biosynthesis
						hsa00250 Alanine, aspartate and glutamate metabolism
						hsa00910 Nitrogen metabolism
						hsa01100 Metabolic pathways
						hsa01200 Carbon metabolism
						hsa01230 Biosynthesis of amino acids
4	*ACSL1* *	rs12641551	100500818	*MIR3945*	10000	x
			100616111	*MIR4455*	100000	x
			2180	*ACSL1*	100000	hsa00061 Fatty acid biosynthesis
						hsa00071 Fatty acid degradation
						hsa01100 Metabolic pathways
						hsa01212 Fatty acid metabolism
						hsa03320 PPAR signaling pathway

						hsa04146 Peroxisome
						hsa04920 Adipocytokine signaling pathway
			643036	*SLED1*	100000	x
5	*SLC22A4, OCTN1* *	rs272893	553103	*LOC553103*	500	x
			6583	*SLC22A4*	500	hsa05231 Choline metabolism in cancer
6	*ELOVL2* *	rs9393903	54898	*ELOVL2*	500	hsa00062 Fatty acid elongation
						hsa01040 Biosynthesis of unsaturated fatty acids
						hsa01212 Fatty acid metabolism
6	*REV3L*	rs3204953	5980	*REV3L*	500	hsa01100 Metabolic pathways
						hsa03460 Fanconi anemia pathway
10	*SCD* *	rs603424	9033	*PKD2L1*	500	x
10	*PNLIPRP2*	rs4751995	5408	*PNLIPRP2*	500	hsa00561 Glycerolipid metabolism
						hsa01100 Metabolic pathways
						hsa04972 Pancreatic secretion
						hsa04975 Fat digestion and absorption
11	*C11orf10*	rs102275	746	*TMEM258*	500	x
11	*FADS1*	rs174546, rs174547, rs174550, rs174570	3992	*FADS1*	500	hsa01040 Biosynthesis of unsaturated fatty acids
						hsa01212 Fatty acid metabolism
11	*FADS2*	rs174546, rs174547, rs174550, rs174570, rs174583	9415	*FADS2*	500	hsa00592 alpha-Linolenic acid metabolism
						hsa01040 Biosynthesis of unsaturated fatty acids
						hsa01212 Fatty acid metabolism
						hsa03320 PPAR signaling pathway
11	*MOGAT2*	rs499974	4135	*MAP6*	100000	x
			80168	*MOGAT2*	100000	hsa00561 Glycerolipid metabolism
						hsa04975 Fat digestion and absorption
			283214	*LOC283214*	100000	x
			84649	*DGAT2*	100000	hsa00561 Glycerolipid metabolism
						hsa01100 Metabolic pathways

						hsa04975 Fat digestion and absorption
			7405	*UVRAG*	100000	x
11	*BUD13*	rs10790162	84811	*BUD13*	500	x
12	*PAH* *	rs1718306	5053	*PAH*	500	hsa00360 Phenylalanine metabolism
						hsa00400 Phenylalanine, tyrosine and tryptophan biosynthesis
						hsa01100 Metabolic pathways
						hsa01230 Biosynthesis of amino acids
14	*PLEKHH1* *	rs7156144	161145	*TMEM229B/ C14orf83*	500	x
14	*SGPP1*	rs7157785	81537	*SGPP1*	100000	hsa00600 Sphingolipid metabolism
						hsa04071 Sphingolipid signaling pathway
			23224	*SYNE2*	100000	x
16	*NTAN1*	rs1135999	23042	*PDXDC1*	500	x
			102724985	*LOC102724985*	500	x
			123803	*NTAN1*	500	x
19	*APOE*	rs7412	348	*APOE*	500	hsa05010 Alzheimer's disease
20	*SPTLC3* *	rs364585	100505515	*LOC100505515*	100000	x
			101929486	*LOC101929486*	100000	x
			55304	*SPTLC3*	100000	hsa00600 Sphingolipid metabolism
						hsa01100 Metabolic pathways
						hsa04071 Sphingolipid signaling pathway

* as annotated by Illig et al 2010 [2] and Shin et al. 2014 [3]

Supplementary Table S8. Suggestive gene-based associations with metabolite traits using SKAT and burden test in EPIC-Potsdam[a]

Metabolite trait	Gene	Nr. of common variants[b]	Nr. of rare variants[b]	p-value SKAT-C[c]	p-value burden-C[c]
PC aa C42:1/PC aa C42:0	*GFRAL*	6	0	1.54E-05	2.15E-06
PC ae C32:1/PC ae C32:2	*PARP2*	3	0	4.95E-06	4.00E-06
PC ae C34:2/PC ae C36:3	*OCA2*	4	0	8.06E-06	1.28E-06
PC ae C40:6	*TMC5*	4	0	1.80E-05	6.01E-06
PC ae C42:4/PC ae C44:4	*OR10J1*	3	1	4.91E-06	2.64E-06
SM (OH) C22:2/SM C24:0	*GPR156*	1	1	6.15E-06	6.15E-06
PC aa C36:3/PC aa C36:5	*TDRD5*	3	2	2.56E-04	1.36E-06
PC aa C36:3/PC aa C38:5	*TDRD5*	3	2	1.49E-03	6.20E-06
SM (OH) C22:2/SM C18:0	*BIN1*	2	0	7.98E-05	1.50E-06
PC aa C32:1/PC aa C34:1	*ZBTB7C*	2	0	2.78E-07	5.24E-05
PC aa C32:1	*ZBTB7C*	2	0	5.14E-06	1.36E-04
PC aa C32:1/ LysoPC a C18:1	*ZBTB7C*	2	0	2.31E-06	2.96E-04
SM (OH) C22:2/SM C16:1	*TFRC*	3	0	1.66E-06	7.53E-04

[a] only sub-cohort; analysis was adjusted for age and sex; [b] rare variants defined as MAF $\leq \frac{1}{\sqrt{2n}}$ ($\leq 0.015_{\text{EPIC-Potsdam}}$);

[c] significance threshold was defined as $P < 0.05$/[number of genes with >1 variants (ranging from 7243 to 7332)]

Supplementary Table S9. Single SNP associations included within gene-based analyses in EPIC-Potsdam and KORA F4

					EPIC-Potsdam					KORA F4					pooled	
**Metabolite trait**	**Gene**	**Chr**	**Exome chip name**	**SNP**	**N**	**Beta[a] (SE)**	**p- value $_{GC}$**	**CAF**	**Coded allele**	**N**	**Beta[a] (SE)**	**p-value**	**CAF**	**Coded allele**	**Beta[a] (SE)**	**p-value**
SM (OH) C22:2/ SM C18:0	*BIN1*	2	exm2254544	rs1060743	2202	0.0001 (0.0325)	0.9982	72.0%	A	2692	0.0021 (0.0276)	0.9385	70.0%	A	0.0013 (0.0210)	0.9522
		2	exm2263907	rs755639	2202	-0.0263 (0.0305)	0.3897	40.9%	C	2692	-0.0227 (0.0256)	0.3757	41.2%	C	-0.0242 (0.0195)	0.2174
SM (OH) C22:2/ SM C16:1	*TFRC*	3	exm376168	rs3817672	2201	0.0330 (0.0299)	0.2704	45.4%	C	2688	-0.0022 (0.0262)	0.9343	43.4%	C	0.0131 (0.0197)	0.5066
		3	exm-rs11915082	rs11915082	2201	0.0264 (0.0302)	0.3822	61.1%	G	2688	-0.0047 (0.0262)	0.8588	59.0%	G	0.0087 (0.0198)	0.6619
		3	exm-rs9859260	rs9859260	2201	0.0078 (0.0304)	0.7969	40.0%	C	2688	-0.0208 (0.0265)	0.4332	38.4%	C	-0.0085 (0.0200)	0.6722
PC aa C42:1/ PC aa C42:0	*GFRAL*	6	exm2270477	rs1007968	2200	0.0263 (0.0300)	0.3858	54.2%	C	2691	-0.0395 (0.0266)	0.1381	54.2%	C	-0.0081 (0.0329)	0.8062
		6	exm556728	rs12199003	2200	-0.0629 (0.0311)	0.0459	61.4%	C	2692	0.0147 (0.0275)	0.5939	60.5%	C	-0.0227 (0.0388)	0.5576
		6	exm556737	rs147652095	2200	-0.0497 (0.1125)	0.6626	98.2%	G	2692	-0.0935 (0.0973)	0.3366	98.1%	G	-0.0748 (0.0736)	0.3097
		6	exm556738	rs115053739	2200	-0.0497 (0.1125)	0.6626	98.2%	G	2692	-0.1046 (0.0982)	0.2866	98.1%	G	-0.0809 (0.0740)	0.2744
		6	exm556756	rs146300118	2200	-0.0497 (0.1125)	0.6626	98.2%	A	2692	-0.1075 (0.0977)	0.2712	98.1%	A	-0.0826 (0.0738)	0.2625
		6	exm556777	rs9370418	2200	0.0320 (0.0333)	0.3415	29.0%	T	2692	-0.0829 (0.0303)	0.0062	26.8%	T	-0.0263 (0.0574)	0.6473

CAF, coded allele frequency; GC, genomic control; SE, standard error

[a] metabolite traits (µmol/L) were ln-transformed, outliers (>4 SD) were removed and metabolite traits were standardized, effect estimates are adjusted for age and sex

Supplementary Table S10. Suggestive gene-based associations including functional exome chip variants with metabolite traits using SKAT and burden test in EPIC-Potsdam[a]

Metabolite trait	Gene	Nr. of common variants[b]	Nr. of rare variants[b]	p-value SKAT-C[c]	p-value burden-C[c]
[Ile+ Leu]/Met	*PCNXL3*	1	1	3.14E-05	3.14E-05
Val/[Ile+ Leu]	*DNAH6*	3	2	3.27E-04	1.35E-05
PC ae C36:2/PC aa C36:2	*OCA2*	2	0	1.64E-05	7.83E-05
PC aa C38:3/LysoPC a C20:3	*COL11A2*	2	0	4.75E-06	2.63E-07
PC aa C38:3/LysoPC a C18:1	*COL11A2*	2	0	4.45E-06	4.67E-07
PC aa C36:3/PC aa C38:5	*DNAJC13*	2	1	2.31E-05	9.60E-05
H1	*OR51Q1*	2	1	8.85E-06	9.62E-06

[a] only sub-cohort; analysis was adjusted for age and sex; [b] rare variants defined as MAF $\leq \frac{1}{\sqrt{2n}}$ (≤ 0.015 $_{\text{EPIC-Potsdam}}$);

[c] significance threshold was defined as P < 0.05/[number of genes with >1 variants (ranging from 1449 to 1492)]

Functional variants were defined based on the Charge annotation list: column “sc_damaging”.

Supplementary Table S11. Single SNP associations included within functional gene-based analyses in EPIC-Potsdam and KORA F4

					EPIC-Potsdam					KORA F4					pooled	
**Metabolite trait**	**Gene**	**Chr**	**Exome chip name**	**SNP**	**N**	**Beta[a] (SE)**	**p-value GC**	**CAF**	**Coded allele**	**N**	**Beta[a] (SE)**	**p-value**	**CAF**	**Coded allele**	**Beta[a] (SE)**	**p-value**
H1	*OR51Q1*	11	exm882803	rs151161477	2192	0.0819 (0.1329)	0.5389	98.79%	G	2676	0.0670 (0.1211)	0.5803	98.92%	G	0.0738 (0.0895)	0.4100
		11	exm882826	rs58283839	2192	-0.1609 (0.1214)	0.1864	98.43%	A	2676	-0.0781 (0.09381)	0.4054	98.21%	A	-0.1090 (0.0742)	0.1419
		11	exm882840	rs2647574	2192	-0.0097 (0.0308)	0.7526	61.13%	C	2676	0.0019 (0.0259)	0.9420	60.70%	G	-0.0029 (0.0198)	0.8823

CAF, coded allele frequency; GC, Genomic control; SE, standard error

[a] metabolite traits (µmol/L) were ln-transformed, outliers (>4 SD) were removed and metabolite traits were standardized, effect estimates are adjusted for age and sex

Supplementary Table S12. Exclusion of top variants from the gene-based analyses for genes (>2 variants) with metabolite traits using SKAT and burden test in EPIC-Potsdam[a]

Metabolite trait	Gene	Chr	Top variant(s)[b]	p-value SKAT-C[c]	p-value burden-C[c]
SM (OH) C22:2/ SM C16:1	*TFRC*	3		**1.66E-06**	**7.53E-04**
			- rs3817672	2.29E-06	0.7711
PC aa C42:1/ PC aa C42:0	*GFRAL*	6		**1.54E-05**	**2.15E-06**
			- rs12199003	1.15E-05	8.89E-07
			- rs146300118	3.17E-05	2.54E-06
			- rs146300118 - rs115053739	0.0008	2.33E-05
			- rs146300118 - rs115053739 - rs147652095	0.1116	0.052
H1	*OR51Q1*	11		**8.85E-06**	**9.62E-06**
			- rs58283839	0.01	0.01

[a] only sub-cohort; [b] excluded from setID file, selection was based on the pooled p-value from single SNP analyses (Supplementary Table S9, 11);
[c] Original p-values in bold, analysis was adjusted for age and sex;

Supplementary Table S13. Gene-based associations of identified genes with diabetes risk using SKAT and burden test in EPIC-Potsdam

Metabolite trait	Gene	Nr. of common variants[a]	Nr. of rare variants[a]	p-value SKAT-C[a]	p-value burden-C[a]
PC aa C42:1/PC aa C42:0	*GFRAL*	6	0	5.79E-01	8.38E-01
SM (OH) C22:2/SM C18:0	*BIN1*	2	0	7.86E-01	7.43E-01
SM (OH) C22:2/SM C16:1	*TFRC*	3	0	4.52E-01	1.32E-01
H1	*OR51Q1*	2	1	6.53E-01	9.83E-01

[a] rare variants defined as MAF $\leq \frac{1}{\sqrt{2n}}$ ($\leq 0.013_{\text{EPIC-Potsdam}}$);

[b] significance threshold was defined as $P < 0.05$

Supplementary Table S14. Identified loci and their function in human metabolism

Locus	Entrez Gene ID*	Name*	Function*
PHGDH	26227	phosphoglycerate dehydrogenase	Enzyme which is involved in the early steps of L-serine synthesis in animal cells. L-serine is required for D-serine and other amino acid synthesis.
CPS1	1373	carbamoyl-phosphate synthase 1	The mitochondrial enzyme encoded by this gene catalyses synthesis of carbamoyl phosphate from ammonia and bicarbonate. This reaction is the first committed step of the urea cycle, which is important in the removal of excess urea from cells. The encoded protein may also represent a core mitochondrial nucleoid protein.
ACSL1	2180	acyl-CoA synthetase long-chain family member 1	The protein encoded by this gene is an isozyme of the long-chain fatty-acid-coenzyme A ligase family. Although differing in substrate specificity, subcellular localization, and tissue distribution, all isozymes of this family convert free long-chain fatty acids into fatty acyl-CoA esters, and thereby play a key role in lipid biosynthesis and fatty acid degradation.
SLC22A4, OCTN1	6583	solute carrier family 22 member 4	Protein is an organic cation transporter and plasma integral membrane protein containing eleven putative transmembrane domains as well as a nucleotide-binding site motif. Polyspecific organic cation transporters in the liver, kidney, intestine, and other organs are critical for elimination of many endogenous small organic cations as well as a wide array of drugs and environmental toxins.
ELOVL2	54898	ELOVL fatty acid elongase 2	ELOVL2 encodes for a transmembrane protein involved in the synthesis of long-chain polyunsaturated fatty acids (PUFA) [Leonard AE et al. Identification and expression of mammalian long-chain PUFA elongation enzymes. Lipids. 2002; 37(8): 733-40.]
REV3L	5980	REV3 like, DNA directed polymerase zeta catalytic subunit	Encodes the REV3L which is a specialized DNA polymerase essential for DNA damage-induced mutagenesis [Gibbs PE et al. A human homolog of the Saccharomyces cerevisiae REV3 gene, which encodes the catalytic subunit of DNA polymerase zeta. Proc Natl Acad Sci U S A. 1998; 95(12): 6876-80.]

SCD	6319	stearoyl-CoA desaturase (delta-9-desaturase)	This gene encodes an enzyme involved in fatty acid biosynthesis, primarily the synthesis of oleic acid. The protein belongs to the fatty acid desaturase family and is an integral membrane protein located in the endoplasmic reticulum.
PNLIPRP2	5408	pancreatic lipase related protein 2	This gene encodes a lipase that hydrolyzes galactolipids, the main components of plant membrane lipids.
FADS1	3992	fatty acid desaturase 1	The protein encoded by this gene is a member of the fatty acid desaturase (FADS) gene family. Desaturase enzymes regulate unsaturation of fatty acids through the introduction of double bonds between defined carbons of the fatty acyl chain.
FADS2	9415	fatty acid desaturase 2	
MOGAT2	80168	monoacylglycerol O-acyltransferase 2	The protein encoded by this gene is an enzyme that catalyses the synthesis of diacylglycerol from 2-monoacylglycerol and fatty acyl-CoA. The encoded protein is important in the uptake of dietary fat by the small intestine. This protein forms a complex with diacylglycerol O-acyltransferase 2 in the endoplasmic reticulum, and this complex catalyses the synthesis of triacylglycerol.
PAH	5053	phenylalanine hydroxylase	PAH encodes the enzyme phenylalanine hydroxylase that is the rate-limiting step in phenylalanine catabolism. Deficiency of this enzyme activity results in the autosomal recessive disorder phenylketonuria.
PLEKHH1	57475	pleckstrin homology, MyTH4 and FERM domain containing H1	NA
SGPP1	81537	sphingosine-1-phosphate phosphatase 1	Sphingosine-1-phosphate (S1P) is a bioactive sphingolipid metabolite that regulates diverse biologic processes. SGPP1 catalyses the degradation of S1P via salvage and recycling of sphingosine into long-chain ceramides
NTAN1	123803	N-terminal asparagine amidase	The protein encoded by this gene functions in a step-wise process of protein degradation through the N-end rule pathway. This protein acts as a tertiary destabilizing enzyme that deamidates N-terminal L-Asn residues on proteins to produce N-terminal L-Asp. L-Asp substrates are subsequently conjugated to L-Arg, which is recognized by specific E3 ubiquitin ligases and targeted to the proteasome.

APOE	348	apolipoprotein E	The protein encoded by this gene is a major apoprotein of the chylomicron. It binds to a specific liver and peripheral cell receptor, and is essential for the normal catabolism of triglyceride-rich lipoprotein constituents. Mutations in this gene result in familial dysbetalipoproteinemia, or type III hyperlipoproteinemia (HLP III), in which increased plasma cholesterol and triglycerides are the consequence of impaired clearance of chylomicron and VLDL remnants.
SPTLC3	55304	serine palmitoyltransferase long chain base subunit 3	The *SPTLC3* gene encodes an isoform of the third subunit of serine palmitoyltransferase (SPT; EC 2.3.1.50), which catalyses the rate-limiting step of the de novo synthesis of sphingolipids.

*According to the NCBI database if not otherwise stated [4]

Supplementary Table S15. List of selected candidate SNPs

SNP	Locus	Chr	Position	Study	Proxy	r²	D’
rs211718	*ACADM*	1	76106675	Illig et al. 2010 [2]			
rs541503	*PHGDH*	1	120208297	Illig et al. 2010 [2]			
rs2286963	*ACADL*	2	211060050	Illig et al. 2010 [2]			
rs715	*CPS1*	2	211543055	Shin et al. 2014 [3]			
rs8396	*ETFDH*	4	159630817	Illig et al. 2010 [2]			
rs12641551	*ACSL1*	4	185767941	Illig et al. 2010 [2]	*(rs2046813)	1	1
rs272893	*SLC22A4, OCTN1*	5	131663062	Illig et al. 2010 [2]	*(rs272889)	1	1
rs329319	*JADE2*	5	133906609	Shin et al. 2014 [3]			
rs9393903	*ELOVL2*	6	11042909	Illig et al. 2010 [2]			
rs603424	*SCD*	10	102075479	Illig et al. 2010 [2]			
rs174547	*FADS1*	11	61570783	Illig et al. 2010 [2]			
rs2014355	*ACADS*	12	121175524	Illig et al. 2010 [2]			
rs4761007	*intergenic*	12	127924890	Shin et al. 2014 [3]			
rs1718306	*PAH*	12	103257308	Shin et al. 2014 [3]			
rs7156144	*PLEKHH1*	14	67979713	Illig et al. 2010 [2]			
rs11158519	*SYNE2*	14	64364585	Illig et al. 2010 [2]			
rs10459872	*C16orf46*	16	81094951	Shin et al. 2014 [3]	*(rs804895)	1	1
rs364585	*SPTLC3*	20	12962718	Illig et al. 2010 [2]	*(rs168622)	1	1
rs11578	*STX16*	20	57253275	Shin et al. 2014 [3]			

* indicates that in the present analysis a proxy SNP in LD of the originally reported GWAS hit (shown in parenthesis) was analyzed

Supplementary Table S16. List of selected metabolite traits (single metabolites, metabolite ratios and factors)

Abbreviation	Biochemical name
**Single metabolites**	
H1	Hexose
Gly	Glycine
Phe	Phenylalanine
SM C16:1	Sphingomyelin C 16:1
PC ae C34:3	Phosphatidylcholine acyl-alkyl C 34:3
PC ae C40:6	Phosphatidylcholine acyl-alkyl C 40:6
PC ae C42:5	Phosphatidylcholine acyl-alkyl C 42:5
PC ae C44:4	Phosphatidylcholine acyl-alkyl C 44:4
PC ae C44:5	Phosphatidylcholine acyl-alkyl C 44:5
PC aa C32:1	Phosphatidylcholine diacyl C 32:1
PC aa C36:1	Phosphatidylcholine diacyl C 36:1
PC aa C38:3	Phosphatidylcholine diacyl C 38:3
PC aa C40:5	Phosphatidylcholine diacyl C 40:5
LysoPC a C18:2	Lysophosphatidylcholine acyl C18:2
**Factors**	
Factor 1 = (0.80×PC ae C32:1) + (0.78×PC ae C32:2) + (0.70×PC ae C34:2) + (0.72×PC ae C34:3) + (0.71×PC ae C36:2) + (0.71×PC ae C36:3) + (0.85×PC ae C40:5) + (0.76×PC ae C40:6) + (0.82×PC ae C42:3) + (0.85×PC ae C42:4) + (0.87×PC ae C42:5) + (0.76×PC ae C44:4) + (0.78× PC ae C44:5) + (0.83×PC ae C44:6) + (0.82×PC aa C42:0) + (0.79×PC aa C42:1) + (0.54×SM C16:1) + (0.57× SM OH C22:2) + (0.41×lysoPC a C17:0)	Factors were derived based on standardized (mean=0; SD=1) single metabolites
Factor 2= (0.55×propionylcarnitine) +(0.66×phe) + (0.61× trp) + (0.66×tyr) + (0.68×val) + (0.66×[ile + leu]) + (0.59×PC aa C32:1) + (0.70×PC aa C36:1)+ (0.65×PC aa C36:3) + (0.76× PC aa C38:3) + (0.72×PC aa C40:4) + (0.71× PC aa C40:5) + (0.44×h1)	Factors were derived based on standardized (mean=0; SD=1) single metabolites
**Ratios amino acids**	
Val/[Ile+ Leu]	Valine/[Isoleucine + Leucine]
[Ile + Leu]/Met	[Isoleucine + Leucine]/Methionine
Tyr/Met	Tyrosine/Methionine
Tyr/Trp	Tyrosine/Tryptophan

Trp/Gln	Tryptophan/Glutamine
Gly/Ser	Glycine/Serine
Ser/Phe	Serine/Phenylalanine
Phe/Arg	Phenylalanine/Arginine
**Ratios Acylcarnitines**	
C3/C0	Propionyl-L-carnitine/DL-Carnitine
**Ratios MUFAs**	
PC ae C32:1/PC ae C32:2	Phosphatidylcholine acyl-alkyl C 32:1/Phosphatidylcholine acyl-alkyl C 32:1
PC ae C34:1/PC ae C32:1	Phosphatidylcholine acyl-alkyl C 34:1/Phosphatidylcholine acyl-alkyl C 32:1
**Ratios PUFAs (C34-C40)**	
PC ae C34:2/PC ae C38:5	Phosphatidylcholine acyl-alkyl C 34:2/Phosphatidylcholine acyl-alkyl C 38:5
PC ae C34:2/PC ae C36:4	Phosphatidylcholine acyl-alkyl C 34:2/Phosphatidylcholine acyl-alkyl C 36:4
PC ae C34:2/PC ae C36:3	Phosphatidylcholine acyl-alkyl C 34:2/Phosphatidylcholine acyl-alkyl C 36:3
PC ae C36:3/PC ae C38:5	Phosphatidylcholine acyl-alkyl C 36:3/Phosphatidylcholine acyl-alkyl C 38:5
PC ae C36:3/PC ae C34:3	Phosphatidylcholine acyl-alkyl C 36:3/Phosphatidylcholine acyl-alkyl C 34:3
PC ae C34:3/PC ae C36:5	Phosphatidylcholine acyl-alkyl C 34:3/Phosphatidylcholine acyl-alkyl C 36:5
PC ae C40:6/PC ae C38:4	Phosphatidylcholine acyl-alkyl C 40:6/Phosphatidylcholine acyl-alkyl C 38:4
PC ae C40:6/PC aa C38:0	Phosphatidylcholine acyl-alkyl C 40:6/Phosphatidylcholine diacyl C 38:0
PC ae C40:6/PC aa C38:6	Phosphatidylcholine acyl-alkyl C 40:6/Phosphatidylcholine diacyl C 38:6
**Ratios PUFAs (C40-C44)**	
PC ae C42:4/PC ae C40:4	Phosphatidylcholine acyl-alkyl C 42:4/Phosphatidylcholine acyl-alkyl C 40:4
PC ae C42:4/PC ae C44:4	Phosphatidylcholine acyl-alkyl C 42:4/Phosphatidylcholine acyl-alkyl C 44:4
PC ae C44:4/PC ae C44:5	Phosphatidylcholine acyl-alkyl C 44:4/Phosphatidylcholine acyl-alkyl C 44:5
PC ae C44:5/PC ae C44:6	Phosphatidylcholine acyl-alkyl C 44:5/Phosphatidylcholine acyl-alkyl C 44:6
PC ae C44:5/PC ae C42:5	Phosphatidylcholine acyl-alkyl C 44:5/Phosphatidylcholine acyl-alkyl C 42:5
PC ae C44:6/PC aa C42:1	Phosphatidylcholine acyl-alkyl C 44:6/Phosphatidylcholine diacyl C 42:1
PC ae C44:6/PC aa C42:0	Phosphatidylcholine acyl-alkyl C 44:6/Phosphatidylcholine diacyl C 42:0
PC aa C32:1/PC aa C34:1	Phosphatidylcholine diacyl C 32:1/Phosphatidylcholine diacyl C 34:1
PC aa C42:1/PC aa C42:0	Phosphatidylcholine diacyl C 42:1/Phosphatidylcholine diacyl C 42:0
PC aa C42:0/PC ae C42:5	Phosphatidylcholine diacyl C 42:0/Phosphatidylcholine diacyl C 42:5

PC ae C42:5/PC ae C40:4	Phosphatidylcholine acyl-alkyl C 42:5/Phosphatidylcholine acyl-alkyl C 40:4
PC ae C40:5/PC ae C42:5	Phosphatidylcholine acyl-alkyl C 40:5/Phosphatidylcholine acyl-alkyl C 42:5
PC ae C40:5/PC aa C42:2	Phosphatidylcholine acyl-alkyl C 40:5/Phosphatidylcholine diacyl C 42:2
PC ae C40:5/PC ae C36:0	Phosphatidylcholine acyl-alkyl C 40:5/Phosphatidylcholine acyl-alkyl C 36:0
PC ae C42:3/PC ae C42:2	Phosphatidylcholine acyl-alkyl C 42:3/Phosphatidylcholine acyl-alkyl C 42:2
PC ae C42:3/PC ae C40:1	Phosphatidylcholine acyl-alkyl C 42:3/Phosphatidylcholine acyl-alkyl C 40:1
**Ratios Sphingomyelins**	
SM(OH)C22:2/SM(OH)C22:1	Hydroxysphingomyelin C 22:2/Hydroxysphingomyelin C 22:1
SM(OH)C22:2/SM C24:1	Hydroxysphingomyelin C 22:2/Sphingomyelin C 24:1
SM(OH)C22:2/SM C24:0	Hydroxysphingomyelin C 22:2/Sphingomyelin C 24:0
SM(OH)C22:2/PC ae C38:2	Hydroxysphingomyelin C 22:2/Phosphatidylcholine acyl-alkyl C 38:2
SM(OH)C22:2/SM C16:1	Hydroxysphingomyelin C 22:2/Sphingomyelin C 16:1
SM(OH)C22:2/SM C18:1	Hydroxysphingomyelin C 22:2/Sphingomyelin C 18:1
SM(OH)C22:2/SM(OH)C14:1	Hydroxysphingomyelin C 22:2/Hydroxysphingomyelin C 14:1
SM(OH)C22:2/SM C18:0	Hydroxysphingomyelin C 22:2/Sphingomyelin C 18:0
SM C16:1/SM C16:0	Sphingomyelin C 16:1/Sphingomyelin C 16:0
SM C16:1/SM C18:0	Sphingomyelin C 16:1/Sphingomyelin C 18:0
SM C16:1/SM C18:1	Sphingomyelin C 16:1/Sphingomyelin C 18:1
SM C16:1/PC aa C28:1	Sphingomyelin C 16:1/Phosphatidylcholine diacyl C 28:1
**Ratios Diacyl-Phophatidylcholines/Lyso-Phosphatidylcholines**	
PC aa C40:4/PC aa C42:6	Phosphatidylcholine diacyl C 40:4/Phosphatidylcholine diacyl C 42:6
PC aa C40:4/PC aa C40:5	Phosphatidylcholine diacyl C 40:4/Phosphatidylcholine diacyl C 40:5
PC aa C40:5/PC aa C42:5	Phosphatidylcholine diacyl C 40:5/Phosphatidylcholine diacyl C 42:5
PC aa C40:5/PC aa C38:6	Phosphatidylcholine diacyl C 40:5/Phosphatidylcholine diacyl C 38:6
PC aa C40:5/PC aa C40:6	Phosphatidylcholine diacyl C 40:5/Phosphatidylcholine diacyl C 40:6
PC aa C40:5/PC aa C36:5	Phosphatidylcholine diacyl C 40:5/Phosphatidylcholine diacyl C 36:5
PC aa C40:5/PC aa C36:3	Phosphatidylcholine diacyl C 40:5/Phosphatidylcholine diacyl C 36:3
PC aa C40:5/PC aa C38:5	Phosphatidylcholine diacyl C 40:5/Phosphatidylcholine diacyl C 38:5
PC aa C36:3/PC aa C36:5	Phosphatidylcholine diacyl C 36:3/Phosphatidylcholine diacyl C 36:5
PC aa C36:3/PC aa C34:3	Phosphatidylcholine diacyl C 36:3/Phosphatidylcholine diacyl C 34:3

PC aa C36:3/LysoPC a C18:1	Phosphatidylcholine diacyl C 36:3/Lysophosphatidylcholine acyl C18:1
PC aa C36:3/PC aa C36:4	Phosphatidylcholine diacyl C 36:3/Phosphatidylcholine diacyl C 36:4
PC aa C36:3/PC aa C38:4	Phosphatidylcholine diacyl C 36:3/Phosphatidylcholine diacyl C 38:4
PC aa C36:3/PC aa C38:3	Phosphatidylcholine diacyl C 36:3/Phosphatidylcholine diacyl C 38:3
PC aa C36:3/PC ae C38:3	Phosphatidylcholine diacyl C 36:3/Phosphatidylcholine diacyl C 38:3
PC aa C36:3/PC aa C38:5	Phosphatidylcholine diacyl C 36:3/Phosphatidylcholine diacyl C 38:5
PC aa C38:3/PC aa C36:5	Phosphatidylcholine diacyl C 38:3/Phosphatidylcholine diacyl C 36:5
PC aa C38:3/PC aa C38:4	Phosphatidylcholine diacyl C 38:3/Phosphatidylcholine diacyl C 38:4
PC aa C38:3/LysoPC a C20:3	Phosphatidylcholine diacyl C 38:3/Lysophosphatidylcholine acyl C20:3
PC aa C38:3/PC aa C36:4	Phosphatidylcholine diacyl C 38:3/Phosphatidylcholine diacyl C 36:4
PC aa C38:3/LysoPC a C18:1	Phosphatidylcholine diacyl C 38:3/Lysophosphatidylcholine acyl C18:1
PC aa C38:3/PC ae C36:2	Phosphatidylcholine diacyl C 38:3/Phosphatidylcholine acyl-alkyl C 36:2
PC aa C38:3/PC ae C38:3	Phosphatidylcholine diacyl C 38:3/Phosphatidylcholine acyl-alkyl C 38:3
PC aa C38:3/PC aa C38:5	Phosphatidylcholine diacyl C 38:3/Phosphatidylcholine diacyl C 38:5
PC ae C36:2/PC aa C32:2	Phosphatidylcholine acyl-alkyl C 36:2/Phosphatidylcholine diacyl C 32:2
PC ae C36:2/PC aa C36:2	Phosphatidylcholine acyl-alkyl C 36:2/Phosphatidylcholine diacyl C 36:2
PC ae C36:2/PC ae C36:1	Phosphatidylcholine acyl-alkyl C 36:2/Phosphatidylcholine acyl-alkyl C 36:1
PC ae C36:2/LysoPC a C17:0	Phosphatidylcholine acyl-alkyl C 36:2/Lysophosphatidylcholine acyl C17:0
PC aa C36:1/LysoPC a C18:1	Phosphatidylcholine diacyl C 36:1/Lysophosphatidylcholine acyl C18:1
PC aa C36:1/PC aa C36:2	Phosphatidylcholine diacyl C 36:1/Phosphatidylcholine diacyl C 36:2
PC aa C36:1/PC aa C34:2	Phosphatidylcholine diacyl C 36:1/Phosphatidylcholine diacyl C 34:2
PC aa C36:1/PC aa C34:1	Phosphatidylcholine diacyl C 36:1/Phosphatidylcholine diacyl C 34:1
PC aa C32:1/LysoPC a C16:1	Phosphatidylcholine diacyl C 32:1/Lysophosphatidylcholine acyl C16:1
PC aa C32:1/LysoPC a C18:1	Phosphatidylcholine diacyl C 32:1/Lysophosphatidylcholine acyl C18:1
PC aa C32:1/PC aa C32:2	Phosphatidylcholine diacyl C 32:1/Phosphatidylcholine diacyl C 32:2
LysoPC a C17:0/LysoPC a C18:0	Lysophosphatidylcholine acyl C17:0/Lysophosphatidylcholine acyl C18:0
LysoPC a C17:0/LysoPC a C16:0	Lysophosphatidylcholine acyl C17:0/Lysophosphatidylcholine acyl C16:0
LysoPC a C18:2/LysoPC a C20:4	Lysophosphatidylcholine acyl C18:2/Lysophosphatidylcholine acyl C20:4
LysoPC a C18:2/LysoPC a C18:0	Lysophosphatidylcholine acyl C18:2/Lysophosphatidylcholine acyl C18:0
LysoPC a C18:2/PC aa C36:2	Lysophosphatidylcholine acyl C18:2/Phosphatidylcholine diacyl C 36:2

LysoPC a C18:2/LysoPC a C18:1	Lysophosphatidylcholine acyl C18:2/Lysophosphatidylcholine acyl C18:1
LysoPC a C20:3/LysoPC a C18:2	Lysophosphatidylcholine acyl C20:3/Lysophosphatidylcholine acyl C18:2

References

1 Kircher, M. *et al.* A general framework for estimating the relative pathogenicity of human genetic variants. *Nature Genetics* **46**, 310-315, doi:10.1038/ng.2892 (2014).

2 Illig, T. *et al.* A genome-wide perspective of genetic variation in human metabolism. *Nature Genetics* **42**, 137-141, doi:10.1038/ng.507 (2010).

3 Shin, S.-Y. *et al.* An atlas of genetic influences on human blood metabolites. *Nature Genetics* **46**, 543-550, doi:10.1038/ng.2982 (2014).

4 *Entrez Gene, National Center for Biotechnology Information, National Library of Medicine (US), Bethesda (MD)*, <http://www.ncbi.nlm.nih.gov/gene> (25.07.2016).
